# High-grade serous tubo-ovarian cancer refined with single-cell RNA sequencing: specific cell subtypes influence survival and determine molecular subtype classification

**DOI:** 10.1186/s13073-021-00922-x

**Published:** 2021-07-09

**Authors:** Siel Olbrecht, Pieter Busschaert, Junbin Qian, Adriaan Vanderstichele, Liselore Loverix, Toon Van Gorp, Els Van Nieuwenhuysen, Sileny Han, Annick Van den Broeck, An Coosemans, Anne-Sophie Van Rompuy, Diether Lambrechts, Ignace Vergote

**Affiliations:** 1 Department of Obstetrics and Gynaecology, Division of Gynaecological Oncology, University Hospitals Leuven, Leuven, Belgium; 2 Department of Oncology, Laboratory of Gynaecologic Oncology, KU Leuven, Leuven, Belgium; 3 VIB Centre for Cancer Biology, Leuven, Belgium; 4 Laboratory for Translational Genetics, Department of Human Genetics, KU Leuven, Leuven, Belgium; 5 Department of Oncology, Laboratory of Tumour Immunology and Immunotherapy, KU Leuven, Leuven, Belgium; 6 Department of Imaging and Pathology, University Hospitals Leuven, Leuven, Belgium; 7 Department of Translational Cell and Tissue Research, KU Leuven, Leuven, Belgium

**Keywords:** Single-cell RNA sequencing, High-grade serous tubo-ovarian cancer, Molecular subtypes, Stromal heterogeneity, Transcriptomic markers, Tumour microenvironment, Prognosis, Overall survival

## Abstract

**Background:**

High-grade serous tubo-ovarian cancer (HGSTOC) is characterised by extensive inter- and intratumour heterogeneity, resulting in persistent therapeutic resistance and poor disease outcome. Molecular subtype classification based on bulk RNA sequencing facilitates a more accurate characterisation of this heterogeneity, but the lack of strong prognostic or predictive correlations with these subtypes currently hinders their clinical implementation. Stromal admixture profoundly affects the prognostic impact of the molecular subtypes, but the contribution of stromal cells to each subtype has poorly been characterised. Increasing the transcriptomic resolution of the molecular subtypes based on single-cell RNA sequencing (scRNA-seq) may provide insights in the prognostic and predictive relevance of these subtypes.

**Methods:**

We performed scRNA-seq of 18,403 cells unbiasedly collected from 7 treatment-naive HGSTOC tumours. For each phenotypic cluster of tumour or stromal cells, we identified specific transcriptomic markers. We explored which phenotypic clusters correlated with overall survival based on expression of these transcriptomic markers in microarray data of 1467 tumours. By evaluating molecular subtype signatures in single cells, we assessed to what extent a phenotypic cluster of tumour or stromal cells contributes to each molecular subtype.

**Results:**

We identified 11 cancer and 32 stromal cell phenotypes in HGSTOC tumours. Of these, the relative frequency of myofibroblasts, TGF-β-driven cancer-associated fibroblasts, mesothelial cells and lymphatic endothelial cells predicted poor outcome, while plasma cells correlated with more favourable outcome. Moreover, we identified a clear cell-like transcriptomic signature in cancer cells, which correlated with worse overall survival in HGSTOC patients. Stromal cell phenotypes differed substantially between molecular subtypes. For instance, the mesenchymal, immunoreactive and differentiated signatures were characterised by specific fibroblast, immune cell and myofibroblast/mesothelial cell phenotypes, respectively. Cell phenotypes correlating with poor outcome were enriched in molecular subtypes associated with poor outcome.

**Conclusions:**

We used scRNA-seq to identify stromal cell phenotypes predicting overall survival in HGSTOC patients. These stromal features explain the association of the molecular subtypes with outcome but also the latter’s weakness of clinical implementation. Stratifying patients based on marker genes specific for these phenotypes represents a promising approach to predict prognosis or response to therapy.

**Supplementary Information:**

The online version contains supplementary material available at 10.1186/s13073-021-00922-x.

## Background

High-grade serous tubo-ovarian cancer (HGSTOC) affects worldwide 239,000 women each year [1] and is typically characterised by a high recurrence rate with poor long-term survival [2, 3]. HGSTOC often becomes resistant to most treatment options, a phenomenon that has been attributed to the extensive inter- and intratumoural heterogeneity in this cancer type [4–7]. Indeed, HGSTOC is characterised by very pronounced patterns of chromosomal instability, which are often highly distinct within the same tumour [7, 8] or between their different metastatic localisations [9], but can often also change during disease progression [10]. In addition, various cellular phenotypes involved in immune activation, hypoxia and extracellular matrix remodelling may determine a tumour microenvironment that favours disease progression and metastases [11–15]—hence contributing to the poor clinical outcome of HGSTOC [12–14, 16, 17].

Several initiatives, such as the Australian Ovarian Cancer Study (AOCS) [18, 19] and The Cancer Genome Atlas (TCGA) [4], have studied HGSTOC by applying conventional bulk gene expression analysis on tumours, identifying 4 molecular subtypes: the mesenchymal, immunoreactive, differentiated and proliferative HGSTOCs. Statistically significant survival differences were found between these molecular subtypes, with better outcome for the immunoreactive subtype [3, 4, 18, 20]. However, cross-study robustness of these signatures remains poor as different subtyping algorithms were used between these studies [19–22] and they were not prospectively validated. In addition, stratification of patients according to these molecular subtypes failed to demonstrate differences in response rates to various therapies in clinical trials [23, 24]. Recently, Schwede et al. [25] demonstrated that assigning individual tumours to one of the molecular subtypes was affected by the stroma admixture. After correction for the stromal content, these molecular subtypes lost their prognostic value, while the stromal gene expression in bulk tumour itself was associated with overall survival. These findings underline the importance of the tumour microenvironment in HGSTOC and highlight the necessity to more accurately explore its heterogeneity and how this contributes to disease progression.

Single-cell RNA sequencing (scRNA-seq) has recently enabled us to explore the transcriptomic diversity of tumours and their stroma at an unprecedented level in various different cancer types (e.g. glioblastoma [26] and lung cancer [27]). With the introduction of unique molecular identifiers (UMI) in droplet-based protocols, thousands of single cells from one biopsy can be analysed simultaneously, reducing amplification errors and facilitating the detection of small populations of cells whose transcriptional programmes are often not detected using bulk RNA sequencing [28]. In ovarian cancer, the potential of this technique was recently demonstrated by Izar et al. [29] who characterised the ascites from ovarian cancer patients by droplet-based scRNA-seq describing several tumour, fibroblast and macrophage subpopulations. Moreover, Olalekan et al. [30] recently applied scRNA-seq to metastatic lesions of the omentum of ovarian cancer patients and used unsupervised clustering of T cells and macrophages to identify high and low T cell infiltration in tumours.

Here, we applied scRNA-seq to several thousands of cancer and stroma-derived cells residing in 7 HGSTOC tumours. This allowed us to reconstruct the phenotypic heterogeneity of the tumour microenvironment in HGSTOC tumours, including non-immune cells, and to identify transcriptional markers specific for each stromal phenotype. Based on these markers, we could estimate the prevalence of these phenotypes in conventional bulk expression datasets, characterise the prognostic value of these stromal phenotypes and evaluate their contribution to the established molecular subtypes.

## Methods

### Patient selection

**Fig. 1:**
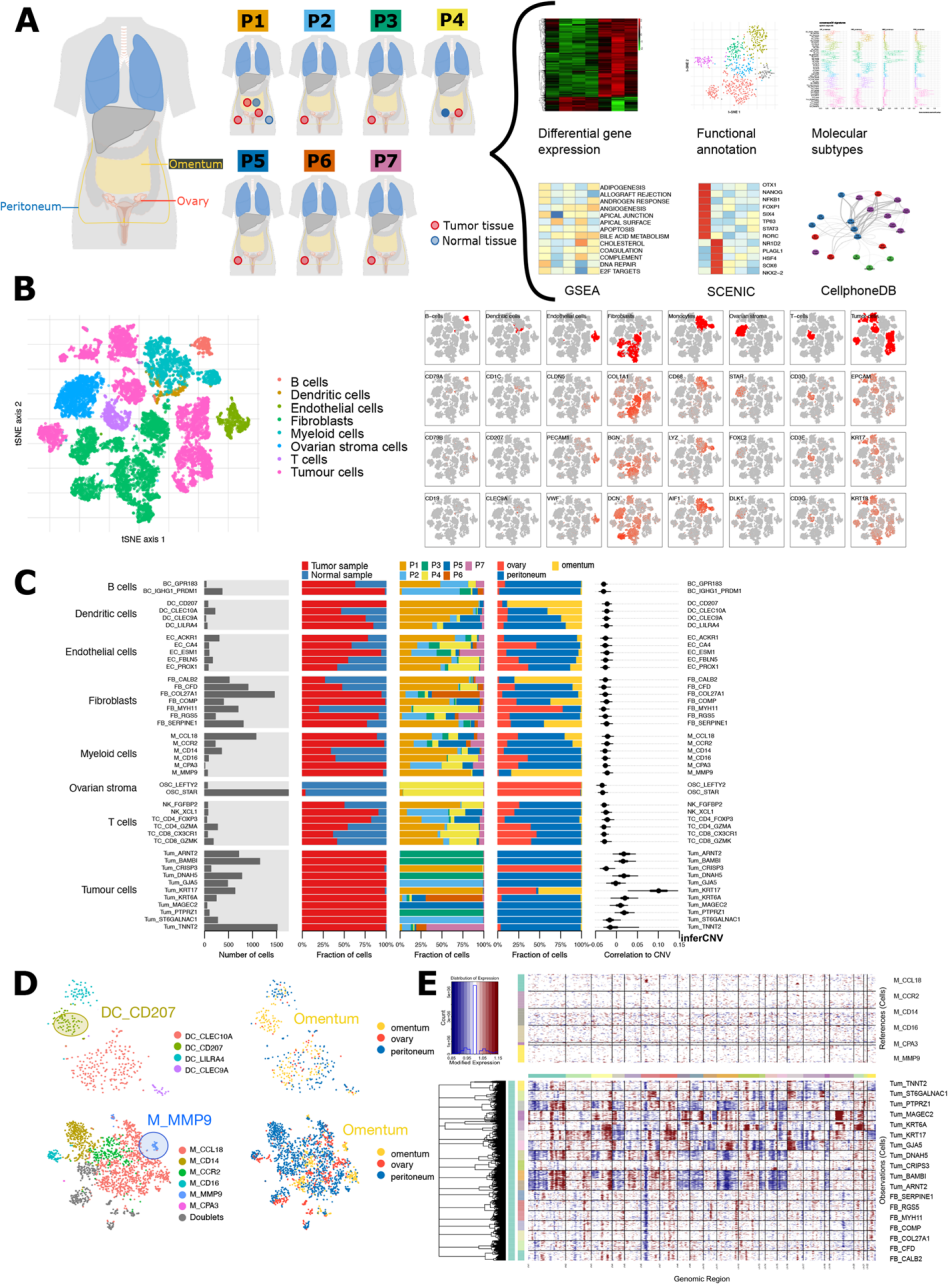
ScRNA-seq-based tumour microenvironment analysis of 18,403 single cells from 7 treatment-naive HGSTOC patients. **A** Schematic overview of the sampling site (ovary, omentum or peritoneum) and tissue type (normal or tumour tissue) of the 12 biopsies from seven treatment-naive patients as well as the analysis workflow. **B** t-SNE representation of all single cells colour-coded for their assigned major cell type (left) and for the expression of three marker genes used for this annotation as indicated on the top row. Marker genes: B cell (*CD79A*, *IGHG3*, *IGKC*), dendritic cells (*CD1C*, *CD1A*, *CLEC9A*), endothelial cells (*CLDN5*, *PECAM1*, *VWF*), fibroblasts (*COL1A1*, *COL1A2*, *BGN*), myeloid cells (*CD68*, *LYZ*, *AIF1*), ovarian stroma cells (*STAR*, *FOXL2*, *DLK1*), T cells (*CD3D*, *CD3E*, *TRAG*), epithelial cancer cells (*EPCAM*, *PAX8*, *CD24*). Dendritic cells remained co-clustered with myeloid cells in the first clustering step, but separated from myeloid cells before further subclustering based on established marker genes (*CLEC9A*, *CD1C*, *CD1A*). **C** Barplot showing for each of the 32 stromal and 11 cancer subclusters (from left to right) the number of cells, tissue type (normal or tumour), their distribution across the 7 patients, their distribution across sampling sites (ovary, omentum and peritoneum) and their correlation to the copy number alteration (CNA) profile of patient 1 using low-coverage whole-genome sequencing. **D** t-SNE visualisation of dendritic cell (up) and myeloid cells (down) subclusters containing tissue-specific cells enriched in the omentum, defined as Langerhans-like dendritic cells (DC_CD207) and lipid-associated macrophages (M_MMP9). **E** CNA profiles of fibroblasts compared to those from the tumour subclusters and monocyte subclusters using inferCNV confirming the CN stable profile of all fibroblast subclusters

Seven patients with ovarian cancer were pathologically diagnosed and treated in the University Hospitals Leuven, Belgium. Written informed consent was obtained from all patients and this study received ethical approval by the ethical committee of University Hospitals Leuven (ML2524/july2013). Fresh biopsies were obtained from these 7 treatment-naive patients during primary cytoreductive surgery (patients P1 and P4) or during diagnostic laparoscopy (patients P2–P3, P5–P7) and consisted of primary ovarian tumour (P1, P4), intraperitoneal metastatic lesions (peritoneum (P1–P3, P5–P7) or omentum (P1)) and normal adjacent tissue (P1 (omental and peritoneal tissue), P4 (ovary)) (Fig. 1A). All samples were analysed by a pathologist experienced in gynaecological pathology (ASVR) and confirmed to be HGSTOC (P1–P2, P4–P7), except patient P3 who presented with a mixed ovarian epithelial carcinoma consisting of clear cell and high-grade serous components. Staging was performed by diffusion-weighted whole-body magnetic resonance imaging (DWI/MRI) [31] according to the FIGO (International Federation of Gynaecology and Obstetrics) classification 2014 [32]. One patient was diagnosed with a stage IC1, 2 patients with stage IIIC and 4 with stage IVB. A concise overview of the clinical characteristics, treatment and response to treatment data is given in Table 1 and Additional file 1: Table S1, respectively.

**Table 1:** Concise overview table of patient characteristics, tissue collection and data metrics of this study

Patient	Histology	FIGO stage*	N of samples	Sample site	Tissue type	Sample_ID°	Total n cells	Median n genes/cell	Median n UMIs/cell	Sequencing saturation
Patient 1	HGSTOC	IIIC	5	Omentum	Tumour	P1TOm	1403	3109	11,544	73.50%
				Omentum	Non-tumour	P1NOm	879	2751	10,090	82.50%
				Peritoneum	Non-tumour	P1NP	1045	1162	2867	93.90%
				Peritoneum	Tumour	P1TP	1249	1192	2958	92.90%
				Ovary	Tumour	P1TOv	1419	3407	12,854	78.20%
Patient 2	HGSTOC	IVB	1	Peritoneum	Tumour	P2TP	2482	2064	7133	81.10%
Patient 3	HGSTOC-CCC	IVB	1	Peritoneum	Tumour	P3TP	3537	2838	9311	63.00%
Patient 4	HGSTOC	IC1	2	Ovary	Tumour	P4TOv	395	2119	7896	87.50%
				Ovary	Non-tumour	P4NOv	3044	1527	5061	67.50%
Patient 5	HGSTOC	IVB	1	Peritoneum	Tumour	P5TP	1343	4610	23,655	74.70%
Patient 6	HGSTOC	IIIC	1	Peritoneum	Tumour	P6TP	2060	4008	20,327	62.90%
Patient 7	HGSTOC	IVB	1	Peritoneum	Tumour	P7TP	1627	1526	5348	63.60%

*HGSTOC* high-grade serous tubo-ovarian carcinoma, *CCC* clear cell carcinoma, *N* number, *UMI* Unique Molecular Identifier

* FIGO stage = disease stage determined by the International Federation of Gynaecology and Obstetrics (FIGO) 2014

° Sample_ID = code attributed to every sample containing 3 elements: (1) patient number (P1–P7), (2) tissue type (T = tumour, N = non-tumour) and (3) sampling site (Om = omentum, Ov = ovary, P = peritoneum)

### Sample preparation for single-cell profiling

Part of the biopsy was embedded in formaldehyde for anatomopathological confirmation, bulk RNA sequencing and/or low-coverage whole-genome sequencing (see below). The other part of the biopsy with a minimal size of 5 mm^3^ was transported in DMEM on ice and digested within 2 h after prelevation to a single-cell suspension. First, the biopsy was rinsed with PBS, minced on ice to small pieces (less than 1 mm^3^) and transferred to 10 ml digestion solution containing DNAse I (Sigma), 0.2% collagenase I/II (Thermo Fisher Scientific) and 25 units dispase (Invitrogen) in DMEM (Thermo Fisher Scientific) [27]. Next, the solution was incubated for 10 min at 37 °C, with manual shaking after 5 min. After incubation, the samples were vortexed for 10 s and pipetted up and down for 1 min using pipettes of descending sizes (25 ml, 10 ml and 5 ml). Next, we added 30 ml ice-cold PBS (pH 7.4) with 2% fetal bovine serum (Thermo Fisher Scientific) and filtered our samples using a 40-μm nylon mesh (Thermo Fisher Scientific). This solution was centrifuged at 300*g* and 4 °C for 5 min. After discarding the supernatant, the cell pellet was resuspended in 2 ml red blood cell lysis buffer and transferred to a 2-ml DNA low bind tube. This solution was incubated for 5 min at room temperature. After a second centrifugation at 120*g* and 4 °C for 5 min, 1 ml PBS containing 8 μl UltraPure BSA (50 mg/ml; AM2616, Thermo Fisher Scientific) was added, followed by filtration of the over Scienceware Flowmi 40-μm cell strainers (VWR) using wide-bore 1 ml low-retention filter tips (Mettler-Toledo). Next, the concentration of remaining viable cells was determined by adding 10 μl of this cell suspension into an immunofluorescence-mediated automated cell counter (Luna FL Cell counter, Logos Biosystems). Cells were kept on ice whenever possible throughout the procedure to avoid dissociation-related artefacts.

### Droplet-based scRNA-seq

Single-cell RNA sequencing libraries were created using the Chromium Single Cell 3’ Library, Gel Bead & Multiplex kit and chip kit (10X Genomics) aiming for 5000 cells per library according to manufacturer conditions. All cells from the same patient were treated with the same master mix and in the same reaction vessel. This droplet-based system uses barcodes (1 for each cell) and unique molecular identifiers (UMIs, 1 for each unique transcript) to obtain a unique 3′-mRNA gene expression profile from every captured cell. All samples were sequenced by the Illumina HiSeq4000 and mapped to the human reference genome (GRCh38) by Cell Ranger (10X Genomics). An overview of the most important metrics including the number of unique molecular identifiers (nUMIs), detected genes and sequencing saturation (76.7% on average) are provided in Table 1. The full metrics, as well as the detailed distribution of the number of cells, genes detected and transcripts (UMI counts) for the major cell types and subtypes can be found in Additional file 2: Table S2 and Additional file 3: Figure S1A.

### Bulk RNA sequencing

Conventional bulk RNA-seq was performed using KAPA stranded mRNA sequencing kits (Roche). After library preparation, all samples were sequenced using the Illumina HiSeq 4000. Reads were trimmed with Trim Galore and mapped using the STAR aligner v2.5 to Ensembl release 90. Transcripts were counted using the summarizeOverlaps function from the GenomicAlignments package v1.22.0 in Bioconductor as described in Love et al. [33].

### Low-coverage whole-genome sequencing

DNA was extracted from one HGSTOC tumour (patient P1) and sequenced genome-wide at low coverage (9 million 51 bp single-end reads) to construct a copy number profile. The reads were mapped to the human reference genome; QDNAseq [34] was used to count reads in preselected, fixed-sized bins and to adjust read counts for mappability and GC content. ASCAT [35] was then used for segmentation and estimation of copy numbers, resulting in a genome-wide copy number profile of the tumour genome for this patient [36].

### Single-cell expression profiling and clustering into cell subtypes

As the identification of (rare) cell type or cell type subclusters from scRNA-seq data depends on the number of cells profiled, we pooled all 12 samples to increase the number of cells analysed [37]. Cell Ranger (10X Genomics) was used to process UMIs (transcripts) and barcodes (cells). For each barcode, the number of associated UMIs was counted. A threshold was set by Cell Ranger at the 10th percentile of the UMI counts; barcodes with this number of UMIs or more were regarded as cells, and barcodes with a lower UMI count were discarded. This procedure retained 20,483 cells across all twelve biopsies (Additional file 2: Table S2). After this step, the Seurat package v2.3.4 in R v3.5.1 was used for all downstream processing as recommended by 10X Genomics. Standard operations as advocated by the Seurat documentation were followed, as described in the following paragraphs.

Genes expressed in < 10 cells were not considered, while cells with < 200 genes expressed were considered low-quality and removed from further analysis. Likewise, cells having > 6000 genes and > 15% of mitochondrial transcripts (indicative of apoptosis) were also removed. In total, we retained an expression matrix of 23,152 genes in 18,403 single cells. This expression matrix was normalised for the total number of transcripts per cell, multiplied by a factor 10,000, and subsequently log-transformed. Variable genes were filtered for genes with an average expression between 0.0125 and 3 and a *z*-score of the logarithmic variance-to-mean ratio > 0.5.

In addition, each cell was scored by Seurat for G2/M and S cell cycle phases based on a gene list for the human genome by the Regev Lab [38]. We corrected for G2/M and S cell cycle scores, the number of UMIs and percentage of mitochondrial transcripts using a linear regression model; the residuals of this model were then scaled and centred to *z*-scores across cells. Regression for cell cycle genes was particularly important for the T cell/natural killer (NK) cell subcluster. Indeed, without regression, a proliferating T cell subcluster containing a heterogeneous mixture of different T cell and NK cell phenotypes, including 2 types of NK cells as well as different CD4+ and CD8+ T cell phenotypes (Additional file 3: Figure S1B–F), was identified.

Next, principal component analysis (PCA) was applied to this rescaled data matrix. The number of informative principal components (PC) covering the highest variance in the dataset was set to 20 based on an elbow plot of the first 40 principal components (Additional file 3: Figure S2A). Similar to others [27, 39–42], we clustered the 20 PCs based on a shared nearest neighbour graph-based clustering method implemented in Seurat, which caters for both small and large populations of cells and is optimised in terms of computing requirements for information-dense, large datasets as the one we generated here. Furthermore, a previous comparative analysis by our research group identified the Seurat clustering method to have high concordance with other clustering methods [27]. Next, clusters were calculated by the Seurat FindClusters function and visualised using t-distributed stochastic neighbour embedding (t-SNE) dimensional reduction method with a default perplexity of 30. Differential gene expression (DGE) analysis was performed at various cluster resolutions by the Wilcoxon rank sum test. The resolution was set at 0.35, because at this resolution cell types that were expected to be identified based on previous studies [27, 39, 43, 44] were effectively recovered, while at lower resolution, they were lost. These cell types were annotated based on the uniform expression of marker genes across the cluster. A full list of these maker genes with Entrez Gene ID and PMID can be found in Additional file 4: Table S3. With this resolution, we were able to distinguish epithelial/cancer cells (TUM), endothelial cells (EC), ovarian stroma cells (OSC), fibroblasts (FB) and the common immune cells (T cells (TC), B cells (BC), myeloid cells (M)) while dendritic cells (DC) still remained co-clustered with myeloid cells. Therefore, DCs were first separated from myeloid cells based on established marker genes (*LILRA4*, *CXCR3*, *CLEC9A*, *CD1C*, *CD1A*, *CD207*) [39] and then pooled together for further subclustering. Robustness of this clustering strategy was confirmed by calculating the Normalised Mutual Information (NMI) [45] and Adjusted Rand Index (ARI) [46] between the clusters using different PCs, resolutions and K values (lower and higher). Both indices quantify the cluster concordance from 0 to 1, with 0 indicating random clustering and 1 perfect concordance.

The cells of each major cell type were then merged and reclustered by repeating the previously described pipeline. The number of variably expressed genes was recalculated for each cell type with the same cut-offs for normalised expression and quantile normalised variance. The G2/M and S cell cycle scores, together with number of UMIs and percentage of mitochondrial transcripts, the patient, interferon scores (BROWNE_INTERFERON_RESPONSIVE_GENES in the Molecular Signatures Database MSigDB v6.1) and the sample dissociation-induced gene signatures [47] were regressed out while rescaling the matrix. Similar to the initial clustering, the choice of the number of principal components and optimal resolution for each cell type was guided by elbow plots and the expression of marker genes of known cell phenotypes in the DGE analysis respectively. The new clusters obtained after reclustering the major cell types are referred to as “subclusters” or, when referring to their function, as “cell phenotypes”.

Shannon indices [48] were calculated to score patient and sample bias in the subclusters, with a low score indicating dominance of a sample/patient in a subcluster and a high score indicating a more even distribution of samples/patients in the subclusters.

To correct for potential batch effects in the fibroblasts, we applied 2 additional alignment methods, i.e. canonical correlation analysis (CCA) [49, 50] and Single-CEll regulatory Network Inference Clustering (pySCENIC). The selection of CCA dimensions or canonical correction vectors (CC = 11) for subspace alignment was guided by the CC bicor saturation plot (MetageneBicorPlot function in Seurat) as recommended, and cluster resolutions were determined similar to the PCA-based approach described above. PySCENIC [51], on the other hand, analysed cells based on the shared activation of gene regulatory networks. PySCENIC clusters were obtained by Ward clustering of Jaccard distances between binarised AUC scores of the pySCENIC algorithm. Shannon indices for PCA-, CCA- and pySCENIC-aligned fibroblasts were compared to choose the optimal alignment for fibroblast downstream analysis.

Doublet subclusters, i.e. subclusters harbouring cells from different major cell types, were identified on a subcluster level based on the simultaneous expression of marker genes from different cell types and were therefore excluded.

Finally, we repeated the normalised mutual information analysis to score subcluster robustness by varying the numbers of PCs (adding or subtracting up to 2 PCs) or resolution (5 or 10% more or less than the selected value). We did not perform this analysis on fibroblasts as a CCA-based alignment was used, nor on B cells for which we deliberately chose to identify only the 2 major subtypes (memory and plasma cells) as further subclustering was not meaningful given the low number of cells (see “Results”).

### Correlation of subclusters to copy number aberration (CNA) profile and inferCNV

Copy number aberrations in single cells were estimated in two ways. First, the tumour copy number profile of patient P1 obtained by low-coverage whole-genome sequencing was used as a reference to compare copy number patterns detected in our single cells. We identified which genes were contained in the copy number segments and correlated gene expression to the copy number of that segment. For this purpose, genes with overall low expression across our single cells were excluded (using the 5th percentile as a threshold), and each gene was standardised by calculating *z*-scores across cells. The Spearman rank order correlation coefficient between *z*-scores of genes and the copy number of the associated segment was calculated. Tumour cells are expected to have overall higher expression in genomic regions with copy number gains and overall lower expression in regions with copy number losses [52]; as such, this correlation is taken as an indication that a cell would be a tumour cell.

As a second, alternative approach, we applied inferCNV version v1.0.3. For inferCNV, 50 cells per subcluster were pseudorandomly chosen; raw expression data of these cells was used as input matrix. Cells previously annotated as monocytes were used as a reference population to infer copy number profiles. The inferCNV cutoff parameter was set to 0.1, and parameters denoise, cluster_by_groups and HMM were set to TRUE.

### Gene set enrichment analysis

To characterise the 43 obtained subclusters, we performed single-sample gene set enrichment analysis (ssGSEA) of the 50 hallmark gene sets of MSigDB (Molecular Signatures Database) [53, 54] for each single cell. Similarly, we performed ssGSVA analysis of the metabolic pathway signatures as listed by Gaude and Frezza [55]. Genes that occur in multiple signatures were excluded for both sets of signatures.

### Analysis of gene regulatory networks

To detect transcription factors driving these 43 different subclusters, we applied Single-CEll regulatory Network Inference and Clustering (SCENIC) using the pySCENIC package [51]. SCENIC identifies regulons—gene sets that are co-expressed with known transcription factors—by cis-regulatory motif analysis. By scoring and comparing the activity of these regulons in each cell, we were able to cluster cells according to their active gene regulatory networks. Finally, we evaluated ssGSEA scores for metabolic and MSigDB hallmark signatures to investigate the molecular pathways active in each regulon subcluster.

### Selection of subcluster-specific transcriptomic markers

Within-cell-type marker genes for each of the subclusters were generated by Seurat using the FindAllMarkers function (based on Wilcoxon rank sum test, restricting genes to log fold change ≥ 0.25 and *p* value ≤ 0.01). We continued filtering these within-cell-type marker genes by comparing them across the other subclusters. Expression of each candidate marker gene of a subcluster was compared to the subcluster with the second highest expression for that marker gene; a Wilcoxon rank sum test was used to select genes based on a *p* value ≤ 0.01, Benjamini-Hochberg-adjusted *p* value ≤ 0.05, and log fold change ≥ 0.25. To further exclude genes with potential background expression outside of the intended subcluster, we studied the distribution of the detection rate of each TM across the intended subcluster, across the subcluster with the 2nd highest expression as well as the mean detection rate of each TMs across all subclusters. We applied 3 additional criteria to select TMs. First, a marker gene had to be representative for the majority of cells belonging to that subcluster. Therefore, we added the restriction that a marker gene must be expressed (at least 1 UMI present) in > 40% of the cells of the subcluster. Next, we removed genes exhibiting high expression in another subcluster, though to a lesser extent, as well as genes with a substantial detection rate across a large number of other subclusters. We therefore added a second and third criteria, i.e. a marker gene must be expressed/detected (at least 1 UMI present) in < 50% of the cells in the subcluster with the 2nd highest expression of that marker gene, and the median of the detection rates of this marker gene in all subclusters must be < 10%. A candidate gene fulfilling all the abovementioned criteria was considered a subcluster-specific transcriptomic marker (TM) for further analyses.

We scored subcluster TMs in 6 cohorts of HGSTOC bulk expression datasets (Table 2), by creating a matrix of subcluster TM genes (rows) by samples (columns). For each row, the *z*-scores of samples within the cohort were calculated, and then averaged per sample for all TMs of a subcluster, resulting in a subcluster-specific *z*-score (SSZ score). This SSZ score was then used as an independent variable in survival analyses.

**Table 2:** List of the publicly available microarray expression datasets included for the survival analysis

Dataset	GEO accession	Microarray platform	Sample size	FIGO Stage IV	Optimal debulking
Bentink et al. [56]	E.MTAB. 386	Illumina HumanRef-8 v2.0 beadchip	128:128:42	15%	78%
Bonome et al. [57]	GSE26712	Affymetrix HG-U133A Array	185:185:46	20%	49%
Ferriss et al. [58]	GSE30161	Affymetrix HG-U133A Gene Chip array	58:47:38	9%	41%
Tothill et al. [19]	GSE9891	Affymetrix HG-U133 Plus 2.0	260:235:42	9%	61%
TCGA [4]	TCGA	Affymetrix HT HG-U133A	510:503:44	15%	73%
Konecny et al. [20]	Mayo Clinic	Agilent Whole Human Genome 4x44K Expression Arrays	382: 369:41	22%	77%

Six whole transcriptome studies with at least 40 patients were evaluated. Exclusion of samples with missing values for age, stage, histology, grade, debulking status and survival data resulted in 1467 patients for downstream analysis. Sample size column provides the number of available samples: number of included samples: median survival time in months

We implemented xCell [59] to examine the enrichment of a certain cell phenotype in bulk RNA data. We mimicked the xCell pipeline as described by Aran et al. [59], using the normalised expression matrix of our subclusters instead of cell lines as main input. We determined the 10%, 25%, 33%, 50%, 67%, 75%, and 90% percentiles of expression for each gene in each subcluster and compared for each subcluster the difference in expression between the 10% percentile of that subcluster and the highest of the 90% percentiles of all other subclusters; the same was done for the 25–75%, 33–67% and 50–50% percentile pairs. Additionally, we evaluated the same comparisons, but with the second and third largest percentile of all other subclusters. Based on these repeated comparisons, we filtered genes that pass a certain threshold; we used the values 0, 0.10, 0.50 and 0.80 as thresholds for the differences, chosen based on the observed range of differences. Genes that pass such a threshold were recorded as a candidate signature for that subcluster. Only candidate signatures containing 8 to 200 genes were retained. As a result, we obtained for each subcluster a large number of candidate signatures (648 in total), which were then tested with ssGSVA. The top-3 candidate signatures for each subcluster were selected based on the *t*-statistic of the ssGSVA scores for each subcluster and constituted our final subcluster signatures. ssGSVA scores for the top-3 signatures were always averaged to obtain a subcluster score. However, as described in the xCell paper, a series of operations followed to transform obtained ssGSVA scores of the selected top-3 subcluster signatures. First, we in silico generated dilutions of subclusters by pseudorandomly mixing single cells of a subcluster with single cells of all other subclusters in 11 concentrations ranging from 0.8 to 25%, with 10,000 random cells per mixture, and 3 replicates of each concentration. We calculated the average ssGSVA scores for the top-3 signatures in each of these artificial mixture samples. The obtained scores were shifted and rescaled to a value between 0 and 1 to enable application in other datasets; we did this based on the 0.01 and 0.99 quantile as outer bounds instead of the minimum and maximum value for robustness. The averaged, shifted and rescaled scores were then modelled as a function of the known concentrations in the mixture; the learned parameters of the power function as described in the xCell paper were used to calibrate future samples. Lastly, the interference caused by closely related cell types was corrected using what the xCell paper describes as spillover compensation. A spillover matrix was constructed based on artificial mixtures of 10,000 single cells, with 25% of cells selected from a target subcluster and 75% of non-target subclusters. This spillover matrix enabled us to estimate concentrations of subclusters in a future sample as a constrained non-negative linear combination of the simulated mixture samples; for a detailed explanation, we refer to the description of this algorithm in the original paper [59].

### Meta-analysis of the prognostic effect of subclusters in 6 HGSTOC cohorts

SSZ scores and xCell scores were evaluated in 6 cohorts of publicly available, annotated bulk expression datasets, covering 1467 high-grade (grade 2 or 3) serous tubo-ovarian carcinoma patients (TCGA [4], Bonome et al. [57], Tothill et al. [19], Bentink et al. [56], Ferriss et al. [58] and Konecny et al. [20]), see Table 2. Other histology, including grade 1 serous, clear cell or endometrioid carcinoma, was excluded.

Expression data and clinical annotation were retrieved from the curatedOvarianData package in R [60] (Bioconductor, doi: 10.18129/B9.bioc.curatedOvarianData); furthermore, the cohort by Konecny et al. [20] was extended as described by Way et al. [61] (Zenodo.org, 10.5281/zenodo.32906), and the clinical data of the TCGA cohort were updated based on Liu et al. [62]. HGSTOC samples with incomplete clinical information on histopathology, grade, residual disease and FIGO stage at diagnosis were excluded.

For each of the cohorts and each of the subclusters, a Cox proportional hazards regression model of overall survival was implemented with the subcluster score (either SSZ score or xCell), FIGO stage, debulking status (residual disease) and age as independent variables. The coefficient for subcluster score and its standard error were used as input for meta-analysis with the rma function of the metafor package in R with parameter method set to REML. The subcluster-specific OS hazard ratios (HR) and *p* values were calculated and corrected for multiple testing by the Benjamini & Hochberg (BH) method. To visualise this effect of subcluster scores on overall survival, we also plotted 3 Kaplan-Meier curves for the pooled cohorts of patients, stratified by the tertiles of SSZ scores of a subcluster. Both the median survival difference in days and log-rank test between the third and first tertile are given as an illustration.

Finally, to exclude the detection of random effects, we performed an iterative analysis by repeating this meta-analysis of the 6 Cox proportional hazard regression analyses including the SSZ scores of the 500 random gene sets of similar sizes for each subcluster (42 × 500 gene sets). Age, FIGO stage and residual disease were covariates. Only in 2.4% of iterations, the same or larger number of significant subclusters was obtained after Benjamini-Hochberg correction.

### Assignment and scoring of molecular subtypes

We used the ConsensusOV package (Bioconductor, doi:10.18129/B9.bioc.consensusOV) as described by Chen et al. [22] to obtain molecular subtype labels for the 1467 bulk samples from the publicly available cohorts and confirm the prognostic effect of the different molecular subtypes in our study cohort. Next, we applied the ConsensusOV algorithm to our single-cell expression matrix and used boxplots to explore the distribution of the 4 molecular subtype scores across all cells from each subcluster. The 4 scores were then used to estimate the relative enrichment or depletion of that subcluster across each molecular subtype signature and to better understand the tumour microenvironment of the four different molecular subtypes.

### Receptor-ligand analysis with CellPhoneDB

For the CellPhoneDB algorithm, we pseudorandomly selected, for individual patients, 2000 cells per subcluster, or the full set if less than 2000 cells were available. For each patient, we ran CellPhoneDB v2.1.1 using its statistical method and 1000 iterations. Cells were labelled with their major cell type annotation for a first analysis, and then with their cell subcluster annotation for subsequent analyses. To additionally make a comparison between molecular subtypes, we also selected samples of patients P1, P2, P5 and P6, because bulk RNA-seq samples of these patients were annotated as respectively proliferative, differentiated, immunoreactive and mesenchymal by the ConsensusOV algorithm; other patients were excluded from this sub-analysis because their molecular subtype annotation was more ambiguous. After running CellPhoneDB, we counted the number of significant interactions (in both directions) between cell types or subclusters.

## Results

### Multi-site sequencing identified tissue-specific stromal cell subtypes

We applied scRNA-seq to 12 biopsies from either ovarian (*n* = 3), peritoneal (*n* = 7) or omental tissue (*n* = 2), collected from 7 treatment-naive patients with HGSTOC and obtained 18,403 cells with high-quality transcriptomic data (Table 1, Fig. 1A). After normalisation, principal component analysis (PCA) was performed using 2766 variably expressed genes to assign all cells to different clusters. After defining the optimal number of principal components (PC = 20, Additional file 3: Figure S2A) and resolution (R = 0.35; see “Methods”), cells were divided in 21 clusters representing 8 major cell types based on canonical marker gene expression across these clusters (Fig. 1B), including epithelial cancer cells, myeloid cells, dendritic cells (DCs), T cells (TCs), B cells (BCs), fibroblasts (FBs), endothelial cells (ECs) and ovarian stromal cells (OSCs). The gene list used for cell type annotation was added to Additional file 4: Table S3.

Interestingly, natural killer (NK) cells and mast cells were scarce and were only identified after subclustering of the T cells and myeloid cells respectively (Additional file 3: Figure S2B). Although we also profiled omental tissue, mature adipocytes were not identified presumably due to their large size, high buoyancy and the fact that they can easily rupture during droplet formation [63]. We confirmed the robustness of our clustering settings by calculating an average Normalised Mutual Information (NMI) of 0.99 while varying the number of PCs, K value and resolution (Additional file 3: Figure S2C). NMI values in function of these parameters are listed in Additional file 5: Table S4.

Next, we reclustered the cells of each major cell type into subclusters using the same strategy, performing 8 individual PCAs (1 for each major cell type), while using for each PCA a different number of variable genes (ranging from 2594 to 8170 genes) and optimal number of informative PCs (ranging from 8 to 29). Optimal resolutions were determined based on marker gene expression of previous studies [27, 64] and ranged from 0.01 for ovarian stromal cells to 3.0 for myeloid cells and T cells. The robustness of subclustering each major cell type was confirmed by calculating the average NMI (ranging from 0.88 to 1.00) at varying numbers of PCs and resolutions (Additional file 5: Table S4; Additional file 3: Figure S2D). We also detected 12 doublet subclusters within the myeloid cells (*n* = 4 subclusters), endothelial cells (*n* = 1), fibroblasts (*n* = 2), cancer cells (*n* = 2) and T cells (*n* = 3) harbouring 748 cells in total. These were excluded from further downstream analysis. The marker gene expression used to identify these doublet subclusters among the abovementioned cell types is illustrated in Additional file 3: Figure S3.

Most T, B, dendritic, myeloid and endothelial cell subclusters contained cells derived from multiple patients and from different anatomic sites (Fig. 1C). However, two subclusters of either dendritic or myeloid cells were predominantly composed of cells from omental tissue (Fig. 1D, Additional file 3: Figure S4A). Based on differential gene expression analysis, these cells were identified as Langerhans-like dendritic cells (DC_CD207) and lipid-associated M2 macrophages (M_MMP9), respectively, two cell types known to be enriched in the omentum [65, 66] (Additional file 3: Figure S4B). Metabolic pathway analysis confirmed this hypothesis showing an active fatty acid metabolism and adipogenesis in both cell subclusters (Additional file 3: Figure S4C). Ovarian stromal cell subclusters contained almost exclusively cells from the non-affected ovary (Fig. 1C, Additional file 3: Figure S4D), i.e. from patient 4 diagnosed with early-stage ovarian cancer (FIGO stage IC1). Two types of ovarian stromal cells, OSC_STAR and OSC_LEFTY2, were identified. OSC_STAR expressed *STAR* and *FOXL2* indicating a role in oestrogen production and the maintenance of granulosa cell identity through the repression of testis-specific genes respectively [67] (Additional file 3: Figure S4E). Pathway analysis showed increased cholesterol metabolism as well as oestrogen and androgen response (Additional file 3: Figure S4F). OSC_LEFTY2 showed increased expression of *LEFTY2* (Additional file 3: Figure S4E), a member of the transforming growth factor family, known to be highly expressed in decidualising human endometrial stromal cells [68]. These cells were therefore classified as a population of endometrial cells, probably localised on the ovary in a context of endometriosis. Indeed, OSC_LEFTY2 did also express *FOXL2*, which is known to be overexpressed by endometrial cells in endometriosis [69].

Most cancer cell subclusters (Tum) also clustered in a patient-specific manner (Fig. 1C, Additional file 3: Figure S5A) but not in a tissue-specific manner. For instance, Tum_KRT17, and to a lesser extent Tum_KRT6A, contained cells originating from both the primary as well as the metastatic sites (omentum and peritoneum) from patient 1 (Additional file 3: Figure S5B).

Finally, we also calculated the Shannon index [48] to evaluate patient or sample bias across the subclusters (Additional file 3: Figure S5C). As expected, Shannon indices were low (≅0) for tumour subclusters and tissue-specific subclusters (DC_CD207, M_MMP9, OSC_STAR, OSC_LEFTY2), indicating dominance of patient or sampling site (omentum vs non-affected ovary) respectively. The other dendritic and myeloid cell subclusters, as well as T, B and endothelial cell subclusters showed high indices (≅ 1.5), confirming the even distribution of samples across these subclusters. However, several fibroblast subclusters had a low Shannon index (≅ 0.5) implying the need for further investigation of possible batch effects.

First, we excluded misclassification of cancer cells in fibroblast subclusters based on copy number alterations (CNA). We confirmed that the CNA profile of fibroblast subclusters differed from the CNA profile of the macrodissected tumour of patient P1 (Fig. 1C), and also contrasted the CNA profile of cancer versus stromal clusters using inferCNV (Fig. 1E). Next, we applied canonical correlation analysis (CCA) and pySCENIC and evaluated batch effects in fibroblasts after this method of clustering (see “Methods”). This resulted in 9 CCA-aligned annotated subclusters of which 2 contained doublets, while pySCENIC identified 9 different subclusters based on their underlying gene regulatory networks (Additional file 3: Figure S5D). Subsequently, Shannon indices were calculated for all three alignments, demonstrating the highest Shannon indices and therefore the lowest patient bias in CCA-aligned subclusters (Additional file 3: Figure S5C). Indeed, Shannon indices of CCA-aligned subclusters were around 1–1.5, which is similar to the other non-tissue-specific PCA-aligned stromal subclusters. Therefore, CCA-aligned fibroblasts subclusters were used for further downstream analysis. For all other major cell types, PCA-aligned subclusters were retained.

### Functional annotation of 32 stromal cell subtypes in HGSTOC.

We then functionally annotated all 35 identified stromal subclusters by differential gene expression analysis for known marker genes. Next, we used the transcriptomic profiles of 49 stromal cell phenotypes generated on various cancer types (including HGSTOC) by scRNA-seq and functionally annotated by Qian et al. [64] to finetune these 35 subclusters. Despite the large difference in number of cells analysed (18,403 vs. 233,591, respectively), 33 of the 35 subclusters showed a comparable transcriptional profile. However, a more detailed comparison let us to merge 4 cell subclusters (because a much smaller subcluster with similar expression as a larger subcluster identified by Qian et al. [64] was found). One additional subcluster of capillary endothelial cells (EC_CA4) was identified by increasing resolution of the endothelial cell subclustering up to 2.0. Two subclusters did not match with a transcriptomic profile described by Qian et al. [64], i.e. FB_COL27A1 fibroblasts and OSC_LEFTY2 granulosa cells, but were considered as separate subclusters, as discussed above. Finally, compared to Qian et al. [64], we did not identify some subclusters because they were rare in ovarian cancer or consisted of too few cells and therefore failed to cluster separately. A comprehensive overview of these subclusters or cellular phenotypes relative to Qian et al. is shown in Additional file 6: Supplementary file 1 and Additional file 7: Table S5.

**Fig. 2:**
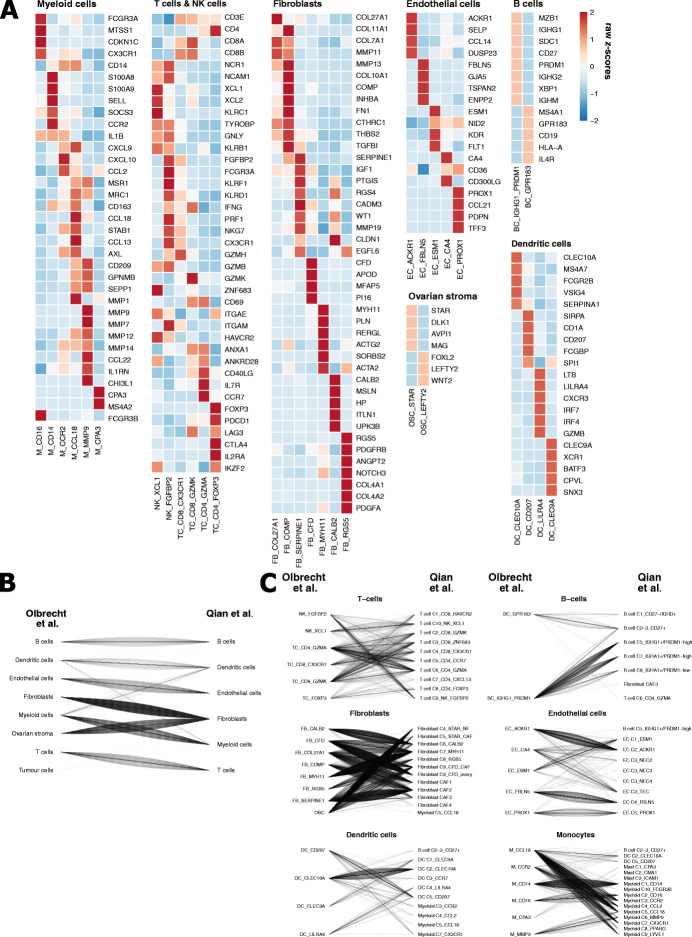
Comparative analysis of subcluster identification between this study and the pan-cancer blueprint. **A** Heatmap illustrating the expression of marker genes for cell type annotation as defined by Qian et al. [64] across all independently clustered myeloid cell, T cell, fibroblast, endothelial cell, B cell, ovarian stroma cell and dendritic cell subclusters. **B**, **C** Subgroup analysis comparing the major cell type (**B**) and cell subtype (**C**) annotation of 8595 cells from 4 patients included in both the pan-cancer blueprint. Line plots illustrate the correspondence of major cell type annotation (**B**) and phenotype annotation (**C**) independently attributed by our analysis (left) and Qian et al. [64] (right). One line represents one cell. As tumour cells were not annotated by Qian et al. [64], only the tumour cells being misclassified to stromal compartments were visualised

Overall, 32 cellular phenotypes were considered for downstream analysis, including 2 BC subclusters, 4 TC subclusters, 2 NK cell subclusters, 5 myeloid and 1 mast cell subcluster, 4 DC subclusters, 5 EC subclusters, 7 FB subclusters and 2 subclusters containing OSC (Fig. 2A). All subcluster metrics (variable genes, PC/CC, resolution) and top 50 genes that were differentially expressed by each subcluster are highlighted in Additional file 8: Table S6. After curation of our subclusters, Shannon indices again confirmed absence of clustering bias (Additional file 3: Figure S5C).

Finally, because Qian et al. profiled 4 out of 7 patients enrolled in this study, we assessed the number of cells with an identical annotation in both studies. Remarkably, 98.5% of cells were attributed to the same cell type (Fig. 2B). This robust overlap in major cell type annotation was confirmed by a normalised mutual information of 0.94. At a subcluster level (see Additional file 6: Supplementary file 1), we obtained an identical annotation for 85.6% of the cells (Fig. 2C) and a normalised mutual information of 0.83 (Additional file 9: Table S7). We therefore consider our clustering strategy to be highly robust and functionally relevant.

### Several cellular subclusters correlate with outcome of HGSTOC

**Fig. 3:**
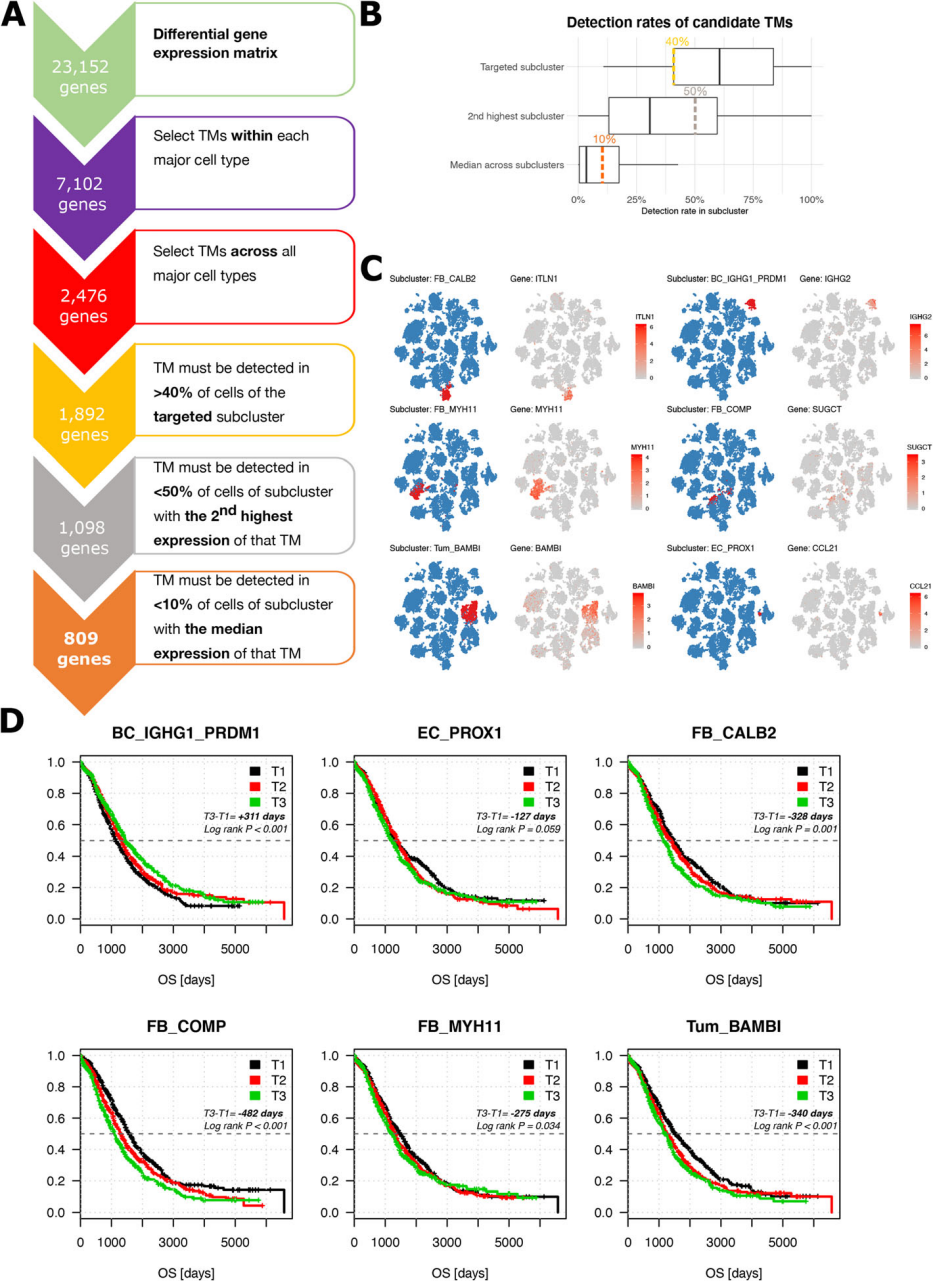
Transcriptomic markers (TMs): filtering strategy and effect on survival. **A** Flowchart illustrating the process used to select 809 TMs. **B** Boxplots illustrating the distribution of the detection rate of each candidate marker gene surviving the selection within and across the major cell types using the Wilcoxon rank sum test (see “Methods”). Shown are the detection rates of each candidate TMs in the targeted subcluster, in the subcluster with the 2nd highest expression of that TMs as well as the median detection rate across all subclusters. Cut-offs for further selection of appropriate TMs were arbitrarily chosen based on this distribution, eliminating all TMs with less than 40% detection rate in the targeted subcluster (yellow), more than 50% detection rate in the subcluster with the 2nd highest expression (grey) and more than 10% median expression across all subclusters (orange). **C** t-SNE of all 18,403 cells visualising the cells classified into the 6 prognostic subclusters based on PCA/CCA alignment on the blue t-SNE as well as the expression of a transcriptomic marker for each of these subclusters across all cells on the grey t-SNE, including *IGHG2* for plasma cells (BC_IGHG1_PRDM1), *CCL21* for lymphatic endothelial cells (EC_PROX1), MYH11 for myofibroblasts (FB_MYH11), *ITLN1* for the mesothelial cells (FB_CALB2), *SUGCT* for the TGF-β-driven cancer-associated fibroblasts (FB_COMP) and *BAMBI* for the cancer cell subcluster Tum_BAMBI. **D** Kaplan-Meier curves for each of the six prognostic cell phenotypes stratifying 1467 patients from 6 public cohorts (TCGA [4], Bonome et al. [57], Tothill et al. [19], Bentink et al. [56], Ferriss et al. [58] and Konecny et al. [20]) in 3 artificial groups based on the presence of a SSZ score in the highest (> 66%, T3), medium (33–66%, T2) and lowest tertile (< 33%, T1) of SSZ scores across the whole dataset. Differences in survival time as well as the log-rank *p* values between group T3 and T1 are indicated. The patients still alive at the time of analysis were censored at the time they were last followed up. In contrast to the meta-analysis used to select these 6 prognostic cell phenotypes, the survival curves and the log-rank *p* values were unadjusted for covariates (age, FIGO stage, residual disease). As a consequence, lymphatic endothelial cells (EC_PROX1) lost their statistical significance (*p* = 0.059)

Next, we selected for each subcluster a unique set of transcriptomic markers (TMs) (Fig. 3A). Based on differential expression, we selected a set of marker genes for each of the subclusters with the FindMarker function in Seurat and then filtered these candidate marker genes by comparing their expression across all the major cell types (see “Methods”), selecting 2476 potential transcriptomic markers. After studying expression of each TMs in the intended subcluster, in the subcluster with the 2nd highest expression of that TM and after considering the mean number of cells expressing the TM across all subclusters, we arbitrarily considered 3 additional criteria to select TMs (Fig. 3B). First, we added the restriction that a TM must be expressed in > 40% of the cells of the respective subcluster, eliminating 584 genes. Secondly, a TM should be expressed in < 50% of the cells of the subcluster with the 2nd highest expression of that TM, further eliminating 794 genes. Lastly, another 289 TMs were excluded as these were detected in > 10% of the cells expressed in the subcluster with the median expression of that TM across all subclusters (Fig. 3A, see “Methods”). Overall, 809 TMs met all the abovementioned criteria, representative for 42 of 43 subclusters (Fig. 3C, Additional file 3: Figure S6). For capillary endothelial cells EC_CA4, we detected 2 TMs (*PRSS23* and *SLC52A3*) none of which survived the additional quantitative filtration. The full list of TMs per subcluster is summarised in Additional file 10: Table S8.

The final number of TMs per subcluster ranged from 1 to 86. Interestingly, 7 of the 10 subclusters with few TMs contained T cells and myeloid cells, which are known to be phenotypically linked to each other [43, 64] (Additional file 10: Table S8), thereby illustrating that it is challenging to select specific TMs for these subclusters. We additionally performed xCell [59] as an alternative gene signature-based deconvolution method. In purified cell lines, the xCell algorithm selected gene signatures for 64 immune and stromal cell types. As these 64 cell types contain non-ovarian cell types, we mimicked the xCell pipeline starting from our 43 single-cell subclusters, resulting in 43 new gene enrichment signatures (see “Methods”).

Next, we used both the 42 sets of TMs and the 43 xCell signatures to estimate the prevalence of each subcluster in expression profiles of bulk samples and to explore which of the subclusters were associated with survival. After z-score transformation of bulk expression datasets derived from 6 published cohorts entailing 1467 HGSTOC patients (Table 2), we first calculated an enrichment score for each subcluster based on our set of TMs and the xCell enrichment signatures (see “Methods”). For the TMs, this score was referred to as the Subcluster-Specific Z-score (SSZ score) and represented the average of all z-scores of the TMs of one particular subcluster. Subsequently, we correlated the SSZ scores and xCell scores with overall survival (OS) of these 1467 HGSTOC patients performing a Cox proportional hazards regression model using FIGO stage, debulking status (residual disease) and age as covariates. SSZ scores and xCell enrichment scores were implemented as continuous values across the 6 individual datasets.

Meta-analyses of the 6 Cox proportional hazards regression analyses (1 per dataset) based on either the SSZ scores or xCell enrichment scores, identified respectively 9 and 14 prognostic subclusters (SSZ scores: Additional file 11: Table S9 and xCell scores: Additional file 12: Table S10). Of these prognostic subclusters, 6 were identified as prognostic by both analyses (Table 3) and therefore retained for further investigation. It concerned mesothelial cells (FB_CALB2), myofibroblasts (FB_MYH11), transforming growth factor ß-driven cancer-associated fibroblasts (FB_COMP), tumour subcluster Tum_BAMBI and lymphatic endothelial cells (EC_PROX1), which had an adverse effect on outcome, while plasma cells (BC_IGHG1_PRDM1high) were associated with improved OS.

**Table 3:** Prognostic subclusters common to both methods after meta-analysis of Cox proportional hazards regression model in 6 cohorts of HGSTOC patients

Subcluster	SSZ score			xCell enrichment score		
	HR [95% CI]	*p* value	BH adj. *p* value	HR [95% CI]	*p* value	BH adj. *p* value
BC_ IGHG1_PRDM1	0.82 [0.74–0.92]	**< 0.001**	**0.007**	0.08 [0.01–0.50]	**0.007**	**0.049**
FB_CALB2	1.47 [1.20–1.81]	**< 0.001**	**0.007**	57.73 [9.34–357.00]	**< 0.001**	**< 0.001**
FB_MYH11	1.25 [1.05–1.50]	**0.014**	0.099	106.52 [14.3–790.0]	**< 0.001**	**< 0.001**
FB_COMP	1.23 [1.10–1.37]	**< 0.001**	**0.007**	4.95 [1.29–19.02]	**0.020**	0.091
TUM_BAMBI	1.47 [1.09–1.99]	**0.012**	0.099	34.11 [4.89–238.10]	**< 0.001**	**0.004**
EC_PROX1	1.29 [1.00–1.65]	**0.049**	0.229	10.35 [1.42–75.13]	**0.020**	0.091

Subclusters significantly affecting OS based on two different scoring methods: subcluster-specific *z*-score (SSZ score; using transcriptomic markers) and the xCell enrichment score (see “Methods”). The meta-analysis hazard ratios (HR) for overall survival, *p* values and the Benjamini-Hochberg-corrected (BH) *p* values are included. Full list of all HR and *p* values for all subclusters in both meta-analyses can be found in Additional file 11: Table S9 (SSZ scores) and Additional file 12: Table S10 (xCell). However, the hazard ratio values are not combined in any way between subclusters or between scoring systems and meta-analyses were conducted per individual subcluster and scoring system. Phenotypes highlighted in the table were chosen based on *p* values of their meta-analyses which are scale independent

After Benjamini-Hochberg correction for multiple testing, BC_IGHG1/PRDM1high and FB_CALB2 remained significant in both meta-analyses, while this was only the case in one of both meta-analyses for FB_MYH11, FB_COMP and Tum_BAMBI. Interestingly, lymphatic endothelial cells EC_PROX1 did not remain significant after multiple hypothesis testing in both analyses. Subcluster-specific hazard ratios (HR), *p* values and false discovery rate-corrected *p* values using the Benjamini-Hochberg for the 6 prognostic subclusters method are highlighted in Table 3. Iterative analysis using 500 random gene sets, confirmed, in only 2.4% of iterations, the same or larger number of significant subclusters obtained after Benjamini-Hochberg correction.

Only TCGA [4] and Tothill et al. [19] reported information on the sampling site (ovary, peritoneum, omentum). Of them, TCGA [4] included almost exclusively ovarian tissue (500/503 samples) while Tothill et al. [19] presented a more diverse cohort, including 161 samples from ovarian tissue and 64 samples from metastatic peritoneal lesion. As a result, no meta-analysis could be performed to investigate to what extent sampling site influenced the survival analysis.

To illustrate the clinical impact of these subclusters, we also performed a Kaplan-Meier analysis illustrating the difference in survival time based on the abundance of each prognostic subcluster using the SSZ scores. In particular, for each prognostic subcluster, we calculated the SSZ score and divided all 1467 HGSTOC patients into the high (> 66%, T3), medium (33–66%, T2) and low (< 33%, T1) SSZ score bins. The difference in survival time is shown in Fig. 3D.

Next, we described the characteristics of the 6 commonly identified prognostic cellular phenotypes.

### Mesothelial cells promote a pro-inflammatory microenvironment in HGSTOC.

**Fig. 4:**
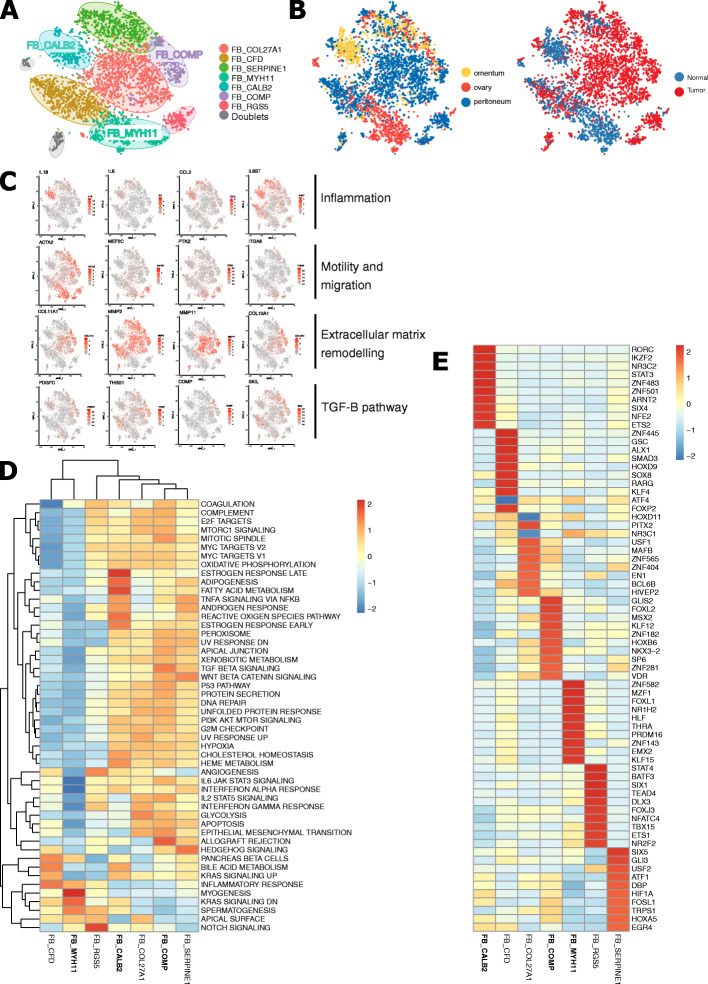
Subclustering of 5950 fibroblasts. **A, B** t-SNE plots showing the annotated fibroblast subclusters generated by CCA-aligned methods (**A**), the fraction of fibroblasts originating from either tumour vs non-tumour tissue or from the different sampling sites (ovary, omentum, peritoneum) (**B**). **C** t-SNEs with gene expression of either mesothelial cells (*IL18*, *IL6*, *CCL2*, *IL6ST*), myofibroblasts (*ACTA2*, *PTK2*, *ITGA8*, *MEF2C*) and cancer-associated fibroblasts expressing EMT-related (*COL10A1*, *COL11A1*, *MMP11*, *MMP2*) and TGF-β-related genes (*COMP*, *THBS1*, *SKIL*, *PDGFC*). **D, E** Heatmap of metabolic activity (**D**) or TF activity (**E**) in fibroblast subclusters

Fibroblasts are well known to promote tumour progression and migration. Especially, cancer-associated fibroblasts (CAFs) facilitate epithelial-to-mesenchymal transition (EMT) and neo-angiogenesis. Remarkably, we observed that, in addition to CAFs (discussed below), two other fibroblast subclusters originating mainly from non-affected tissue (Figs. 1C and 4A–B), i.e. mesothelial cells (FB_CALB2) and myofibroblasts (FB_MYH11), were associated with poor outcome. Mesothelium-derived fibroblasts (FB_CALB2) were characterised by co-expression of *CALB2*, *WT1*, *MSLN* and keratins (*KRT8*, *KRT18*) [70] (Fig. 2A). FB_CALB2 contained cells from all patients and all different anatomic sites, but was enriched in adjacent normal and malignant omental tissue (Fig. 4B). FB_CALB2s expressed high levels of pro-inflammatory cytokines (*IL6* and *IL18*) and IL6-associated genes promoting fibrosis (*COL8A1*, *CXCL16*) and inflammation (*CCL2*, *CXCL1*, *IL6ST*; Fig. 4C). *IL6* is known to promote cell growth, migration, neo-angiogenesis and chemotherapeutic resistance in ovarian cancer [71]. Metabolic pathway analysis showed an upregulation of pathways involved in lipid-metabolism (adipogenesis, bile acid, fatty acid metabolism, cholesterol haemostasis) as well as an activated TNFα NF-κß pathway [72], responsible for the *IL6* production (Fig. 4D). Regulatory analysis with pySCENIC confirmed activation of transcription factor *STAT3*, which is known to interact with *IL6* (Fig. 4E) [73] as well as an upregulation of transcription factors involved in adipocyte differentiation (*SIX4*, *FOSL1*) and fatty acid metabolism (*NKX2-8*) (Fig. 4E).

### Mature IgG-secreting plasma cells promote antitumour activity

**Fig. 5:**
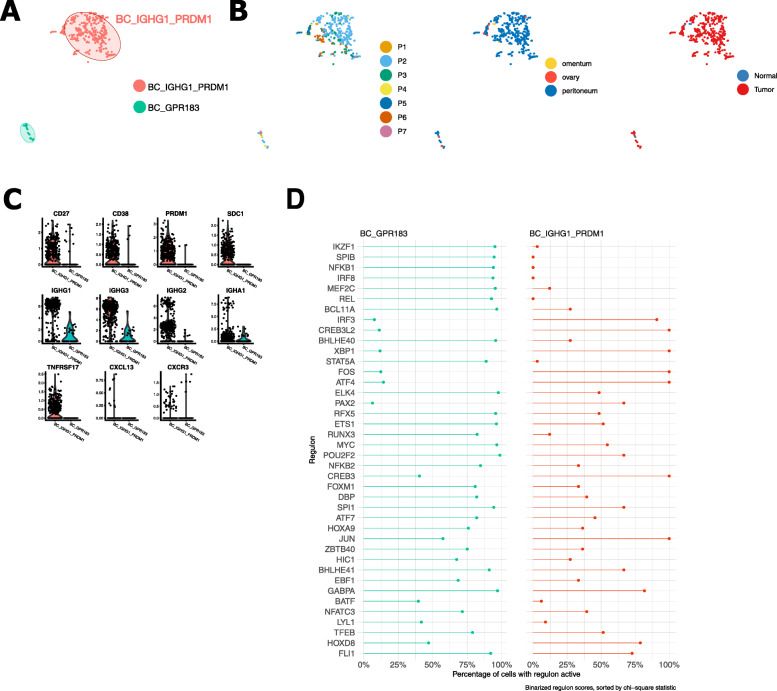
Subclustering of B cells. **A, B** t-SNE plots showing the annotated B cell subclusters generated by PCA-aligned methods (**A**), as well as the fraction of B cells originating from each patient, from the different sampling sites (ovary, omentum, peritoneum) and the different types of tissue (normal vs tumour) (**B**). **C** Violin plots illustrating the expression of maturity markers (*CD38*, *SDC1*, *CD27*, *PRDM1*), immunoglobulins (*IGHG1*, *IGHG2*, *IGHG3*, *IGHA1*) and inflammatory chemokines and receptors (*TNFRSF17*, *CXCR3*, *CXCL13*). **D** Percentage of cells demonstrating transcription factor activity (regulons) across B cell subclusters (pySCENIC)

B cells were present in all patients, although their prevalence varied quite considerably: 70% of B cells were retrieved from patient P2, while patient P1, P4 and P7 together contributed to < 5% of B cells (Fig. 5A, B). It is noteworthy that it has earlier been shown that the proportion of B cells varies widely in HGSTOC tumours [74]. BC_IGHG1_PRDM1^high^ expressed high levels of *PRDM1* (alias *BLIMP1)* as well as plasma cell differentiation markers (*CD27*, *CD38* and *SDC1*) [74, 75] and immunoglobulins (*IGHG1*, *IGHG2* and *IGHG3)* confirming that they represented mature (post-germinal centre) antibody-secreting plasma cells (Fig. 5C). The predominant antibody subtypes in HGSTOC were IgG1 and IgG3. Interestingly, these cells also expressed *TNFRSF17* (B cell maturation antigen) which is essential for the survival of long-lived plasma cells [74]. *TNFRSF17* expression was restricted to plasma cell high tumours regardless of the presence of other tumour-infiltrating lymphocytes (T cells, memory B cells) and linked to an improved survival in ovarian cancer [74]. Transcription factor analysis showed activation of transcription factor *XBP1* (Fig. 5D), required for plasma cell differentiation, immunoglobulin production and plasma cell survival [76, 77].

### Myofibroblasts and TGF-β-driven cancer-associated fibroblasts negatively affect OS

Beside mesothelial cells, 2 other subtypes of fibroblasts containing myofibroblasts (FB_MYH11) and TGF-β-driven cancer-associated fibroblasts (FB_COMP) were linked to a decreased survival.

Myofibroblasts originated either from non-affected ovarian tissue or, to a lesser extent, from peritoneal metastases (Fig. 4B), and were characterised by high expression of *ACTA2* and *MYH11*, two established myofibroblast markers. Among other genes related to myogenesis (*PLN*, *MEF2C* and *CNN1*), these cells expressed a distinct set of integrins (*ITGA7*, *ITGA8*, *ITGA10*) and focal adhesion kinase *PTK2* (Fig. 4C). Release of integrins induces autophosphorylation of PTK2 and regulates cell motility by the cyclic assembly and disassembly of focal adhesion complexes in myofibroblasts [78]. Moreover, although all fibroblasts express *S100A4*, FB_MYH11s showed the highest expression of *S100A4*. S100A4 regulates phosphorylation and filament assembly of myosin II (*MYH11*). Regulatory analysis with pySCENIC demonstrated an upregulation of *MZF1* and *FOXL1* transcription factors (Fig. 4E), indicative for an active myogenesis. Indeed, gene set enrichment analysis (GSEA) confirmed the upregulation of myogenesis and inflammatory response (*AXL*, *SLC7A2*), while apoptosis, hypoxia, IL6/JAK/STAT3 signalling and interferon alpha and gamma response were suppressed (Fig. 4D). Although the TGF-β signalling pathway is often described as a key modulator explaining the malignant potential of myofibroblasts, FB_MYH11s originated mostly from adjacent normal tissue and did not show upregulation of this pathway.

We identified 3 types of CAFs (FB_COL27A1, FB_SERPINE1 and FB_COMP) but only the latter negatively affected OS. FB_COMPs were present in all patients and in all tumour biopsies (Figs. 1C and 4B). As expected, FB_COMP, FB_SERPINE1 and FB_COL27A1 all showed active EMT, as illustrated by high metalloprotease (*MMP2*, *MMP14*, *MMP11*) and collagen (*COL10A1*, *COL11A1*, *COL5A1*, *COL1A1*, *COL27A1*) expression, enabling these cells to degrade the extracellular matrix and escape from the primary tumour site to metastasise (Fig. 4C). *COL1A1*, *COL11A* and *Thrombospondin-1* (*THBS1*) are indeed associated with tumour invasiveness and poor prognosis in ovarian cancer [79]. Despite similarities in gene expression between CAF clusters, metabolic pathway analysis revealed the TGF-β pathway as a key pathway inducing EMT in FB_COMPs (Fig. 4D). This was demonstrated by high expression of TGF-β-associated genes, including *COMP*, *LTBP2*, *SKIL*, *TGFBI*, *PDGFC* and *THBS1* (Fig. 4C). Regulatory analysis demonstrated an activation of *SIX1* (Fig. 4E). *SIX1* induces EMT and is found to be a crucial mediator to the switch of the TGF-β signalling pathway from a tumour suppression to tumour promotion [80]. Furthermore, as expected for CAFs, glycolysis, hypoxia and apoptosis were upregulated (Fig. 4D) [81].

### A clear cell adenocarcinoma-like phenotype associated with poor outcome

**Fig. 6:**
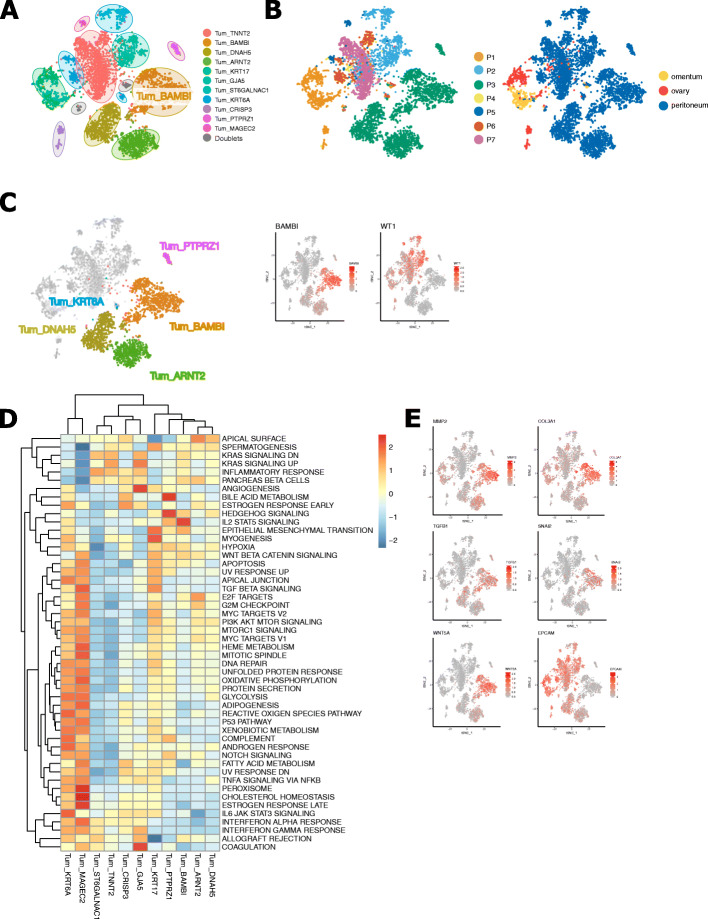
Identification of distinct sets of cancer cells. **A, B** t-SNE plots showing the annotated cancer cell subclusters generated by unaligned PCA methods (**A**) as well as the distribution of cancer cell subclusters across patients and different sampling sites (ovary, omentum, peritoneum) (**B**). **C** t-SNE visualisation of 6 tumour subclusters originating from patient 3 with mixed HGSTOC-clear cell histopathology. Tum_BAMBI, Tum_ARNT2, Tum_DNAH5 and Tum_PTPRZ1 lacked expression of *WT1* suggesting a clear cell origin of these subclusters, while Tum_KRT6A represents the HGSTOC component. **D** Heatmap showing metabolic activity of the tumour subclusters. **E** t-SNEs with marker gene expression indicating an active EMT pathway

All subclusters consisting of malignant cells were mostly patient-specific (Figs. 1C and 6A,B). From the 11 cancer cell subclusters identified in these 7 patients, only one phenotype (Tum_BAMBI) was linked to worse survival. Tum_BAMBI contained the largest number of cells originating from patient P3, who had a mixed high-grade serous tumour with clear cell elements. Based on the absence of *WT1* expression (Fig. 6C), this cluster most likely contained cells from the clear cell component of the tumour [82], which is another histopathological type of epithelial ovarian cancer characterised by a worse prognosis compared to HGSTOCs [83]. Molecular pathway analysis confirmed EMT and IL2/STAT5 signalling in particular to be enriched in Tum_BAMBI (Fig. 6D). This was accompanied by elevated expression of matrix metalloproteases (*MMP2*, *MMP14*), collagens (*COL1A1*, *COL1A2*, *COL3A1*) and classical EMT markers (*TWIST1*, *ZEB1*, *WNT5A* and *SNAI2*), while epithelial markers were downregulated (absence of *EPCAM*) (Fig. 6E).

Importantly, in the current study, bulk RNA cohorts were composed of HGSTOCs only and pure clear cell adenocarcinomas were excluded. Nevertheless, some HGSTOC samples expressed high levels of the TMs expressed by Tum_BAMBI and were correlated with poor outcome. Interestingly, expression of the marker gene *BAMBI*, a TGF-β pseudoreceptor involved in EMT and constitutive IL2/STAT5 signalling, has previously been correlated with tumour growth, tumour invasion and carboplatin resistance in HGSTOC [84, 85]. Overall, this suggests that some HGSTOC tumours can harbour a clear cell-like phenotype [86].

### Lymphatic endothelial cells promote lymph node metastasis in HGSTOC

**Fig. 7:**
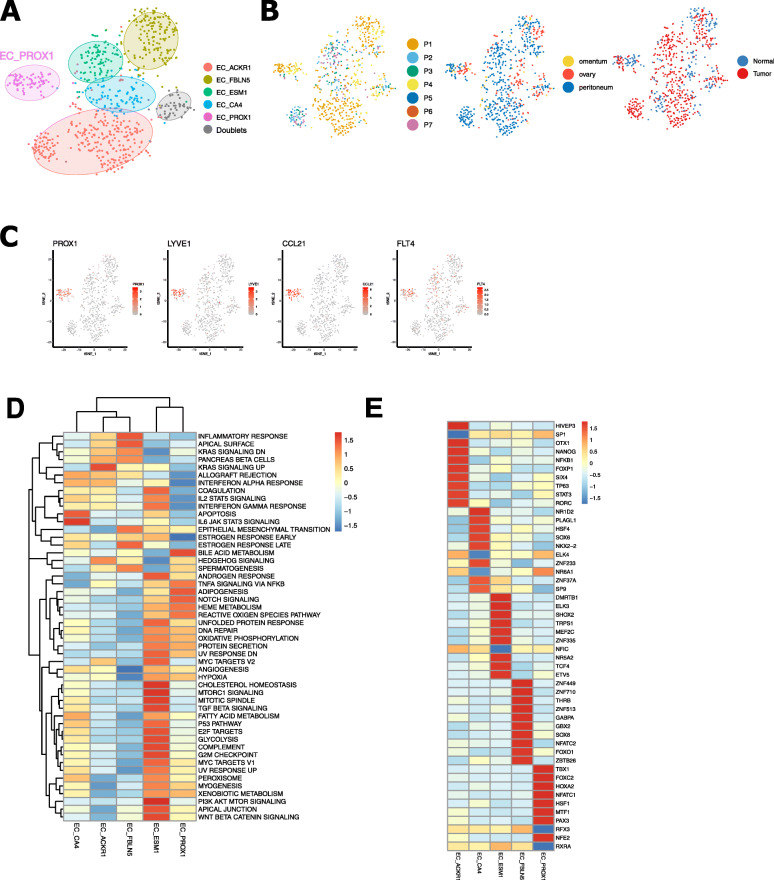
Subclustering of endothelial cells. **A, B** t-SNE plots showing colour-coded the annotated endothelial cell subclusters (**A**) as well as the distribution of the endothelial cells across patients, the different sampling sites (ovary, omentum, peritoneum) and type of tissue (tumour vs non-tumour) (**B**). **C** t-SNE plots illustrating the gene expression of lymphatic endothelial cells (*PROX1*, *LYVE1*, *CCL21*, *VEGFR3*). **D, E** Heatmap of active metabolic pathways (**D**) and transcription factor activity (**E**) in endothelial cell subclusters

The subcluster EC_PROX1 contained cells from all patients and all localisations, both from non-tumour and tumour tissue (Fig. 7A,B). Based on the expression of prospero homeobox protein (*PROX1*), Podoplanin (*PDPN*) and lymphatic vessel endothelial hyaluronan receptor (*LYVE-1*), these cells are considered to be lymphatic endothelial cells. They also expressed *FLT4* (alias *VEGFR3*) known to promote proliferation, differentiation and migration of lymphatic endothelial cells [87, 88] (Fig. 7C). Furthermore, EC_PROX1 highly expressed *CCL21*, a ligand of *CCR7*, important for lymphocyte and dendritic cell trafficking [89] (Fig. 7C). Recently, the interaction between *CCR7-CCL21* was identified as a key paracrine mediator promoting migration of tumour cells towards lymphatic endothelial cells in breast cancer cells, and hence favouring tumour lymph node metastasis [88, 89]. Furthermore, pathway analysis confirmed an upregulation of the TNFα NFκβ pathway which induces enhanced proliferation and migration of lymphatic endothelial cells and can modulate lymphatic metastasis through this particular CCR7-CCL21 axis [90] (Fig. 7D). Besides a high expression of transcription factor *PROX1*, gene regulatory network analysis (pySCENIC) showed an increased activity of *TBX1* (Fig. 7E) which enhances *VEGFR3* expression by binding to an enhancer element in the *VEGFR3* gene and is required for lymphatic vessel development [91].

### Cell phenotypes contributing to the HGSTOC molecular subtypes

**Fig. 8:**
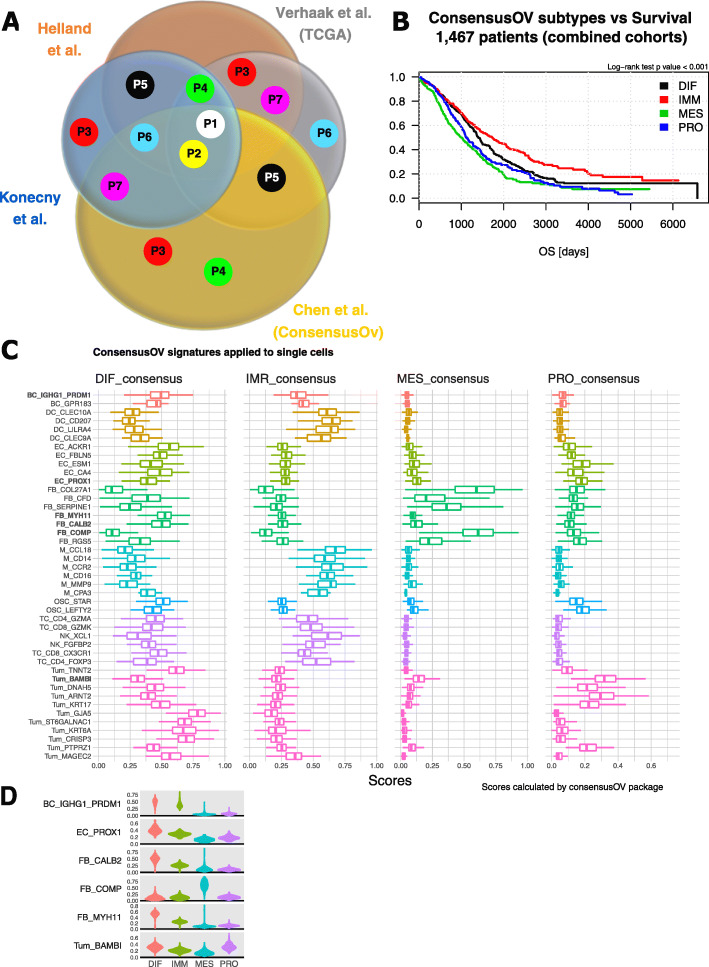
Analysis of the single-cell composition of the four molecular subtypes of HGSTOC. **A** Venn diagram illustration the molecular subtype classification of the 7 patients included in this study, by four different subtyping algorithms using conventional bulk RNA sequencing data. Coloured dots indicate an individual label given to a particular patient. Dots positioned in the four-way intersection, indicate a unique label agreed upon by all subtyping algorithms (patients 1 and 2). Patients 4, 5, 6 and 7 received two different labels, patient 3 three different labels. Subtyping algorithms used were as follows: Helland et al. (Plos One 2011), Verhaak et al. (JCI 2013), Konecny et al. (JNCI 2014), ConsensusOV Chen et al. (CCR 2018). **B** Kaplan-Meier curves indicating the difference in survival time across the 1467 HGSTOC patients included in our reference cohort based on the ConsensusOV algorithm. Results of the log-rank test confirmed the prognostic value of the molecular subtypes (*p* < 0.001). Patients still alive at the time of analysis were censored at the time they were last followed up. Survival curves are unadjusted for covariates and the analysis includes all randomly assigned patients. **C** Molecular subtype scores of our 18,403 single cells calculated by the ConsensusOV package. For all 18,403 single cells the differential gene expression data were used to individually score each cell for the 4 molecular subtype signatures. Global scoring for all cells in one subcluster is visualised by boxplot presentation. Prognostic cell phenotypes were marked in bold. **D** Violin plots showing the enrichment scores of the four molecular ConsensusOV subtypes in each cell of the 6 prognostic subclusters

Subsequently, we used our scRNA-seq data to explore the cellular heterogeneity of the four molecular subtypes underlying HGSTOC. We first assigned a molecular subtype based on bulk RNA-seq data from each of the 7 tumours using 4 previously published molecular subtyping algorithms [20–22, 92]. Only for 2 patients (P1 and P2), the 4 algorithms assigned the same molecular subtype (Fig. 8A), illustrating the lack of agreement between these 4 approaches to subtyping. We chose the ConsensusOV algorithm [22] to determine the molecular subtypes of our study cohort because this algorithm determines a consensus subtype label uniting the 3 original subtyping methods [20, 21, 92]. Interestingly, using ConsensusOV, we could also confirm the previously reported prognostic effect of the molecular subtypes [3, 4, 18, 20] in the 6 published cohorts entailing 1467 HGSTOC patients (Fig. 8B; *p* < 0.001).

Next, we scored all 18,403 single cells individually for each of the 4 molecular subtype signatures using the ConsensusOV algorithm and illustrated for each of the molecular subtypes the distribution of these scores within each subcluster (Fig. 8C). Subsequently, we compared the distribution of these scores for each subcluster in each subtype, enabling us to interpret relative trends in the enrichment or depletion of a cell subcluster in the four different molecular subtypes. Immune cells scored very low for the mesenchymal and proliferative gene signatures, but, as expected, high for the immunoreactive and differentiated signatures. DCs and monocytes in particular expressed genes linked to the immunoreactive subtype, while B cells and T cells showed a similar scoring for immunoreactive and differentiated subtypes (Fig. 8C). Interestingly, only the fibroblasts subclusters (with the exception of FB_MYH11 myofibroblasts and FB_CALB2 mesothelial cells) showed high scores for the mesenchymal subtype, suggesting an important contribution of genes expressed by fibroblasts to identify the mesenchymal subtype in HGSTOC. Myofibroblasts (FB_MYH11) and mesothelial cells (FB_CALB2) on the other hand, as well as endothelial cells showed the highest scores for the differentiated subtype (Fig. 8C).

Interestingly, tumour cells exhibited high scores for either the differentiated or the proliferative molecular subtypes of HGSTOC. Noteworthy, tumour subclusters with high scores for the proliferative subtype (Tum_BAMBI, Tum_KRT17, Tum_DNAH5, TUM_ARNT2 and TUM_PTPRZ1) had relatively low scores for the differentiated subtype, while inversely those ranking high in the differentiated subtype (Tum_MAGEC2, Tum_TNNT2, Tum_GJA5, Tum_ST6GALNAC1, Tum_KRT6A and Tum_CRISP3) were mostly negative for the proliferative subtype. GSEA confirmed the distinct gene expression between both groups of tumour cell phenotypes. Tumour subclusters expressing proliferative genes were enriched for EMT, hypoxia and hedgehog signalling, all contributing to the more aggressive behaviour of proliferative HGSTOC. On the other hand, tumour subclusters with high scores for the differentiated subtype showed an active interferon gamma and interferon alpha response, suggesting, together with high scores in immune cells, an active role of the immune system in differentiated HGSTOC (Fig. 6E). Moreover, by studying the results of our CNA analysis across both groups (Fig. 1C), tumour subclusters scoring high for the proliferative molecular subtypes showed a higher degree of CNV instability while the tumour subclusters more related to the differentiated molecular subtype showed less CNV instability. Identical analyses using the three other methods were added in Additional file 3: Figure S7.

### Cell-cell interactions differing between the HGSTOC molecular subtypes

**Fig. 9:**
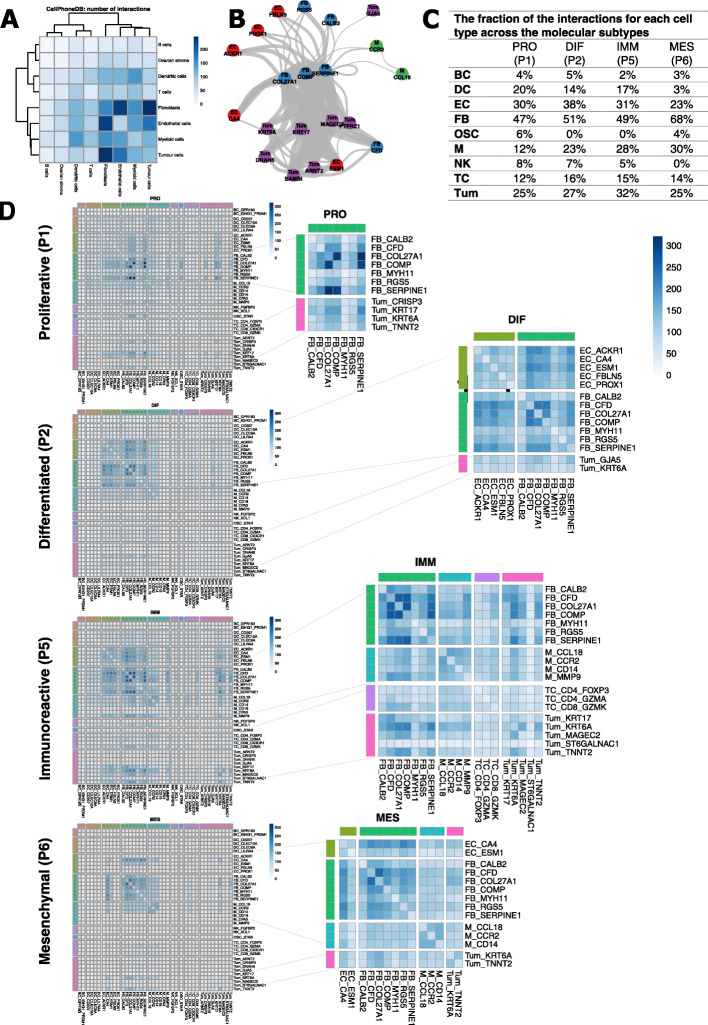
Results of the CellPhoneDB analysis on our scRNA-seq dataset. **A** 81,893 significant interactions were retained based on predicted *p* values for a ligand–receptor complex across two cell clusters, calculated using permutations in which cells are randomly re-assigned to a cluster. The strongest interactions, based on the number of interactions found, were shown between fibroblasts, tumour cells and endothelial cells. **B** Graph network representation of the interactions between subclusters. Only subcluster pairs with more than 170 interactions (i.e. 70% of pairs) are displayed. Edges are weighted by number of interactions. On a subcluster level the strongest interactions are found between FB_COL27A1, FB_SERPINE1 and FB_COMP. **C** Table illustrating the fraction of the interactions involving the different cell major cell types across the molecular subtypes. Dendritic cells showed scarce interactions in mesenchymal HGSTOC (3% compared to 16–24% in the other molecular subtypes) but as only 1 DC was detected in patient 6 the results for DCs in mesenchymal HGSTOC are not reliable. **D** Heatmaps visualising the distribution of the significant interactions between the different cell phenotypes across the different molecular subtypes: patient P1 proliferative HGSTOC, patient P2 differentiated HGSTOC, patient P5 immunoreactive HGSTOC and patient P6 mesenchymal HGSTOC. Zoom-in on the molecular subtype-specific interactions. Intensity of the interactions is measured is scaled by with the number of interactions between those two subclusters

To further investigate how the different cellular phenotypes in HGSTOC interact and how this differs across the 4 molecular subtypes, we applied CellphoneDB vs2.0 [93], a pipeline which calculates the interactions between groups of single cells (i.e. the subclusters; see “Methods”). Assessment of all cells retrieved from our 7 patients retained 81,893 significant interactions between the different clusters (Additional file 13: Table S11). Most interactions were detected between fibroblasts, tumour cells and endothelial cells (Fig. 9A), and, in particular, the CAF FB_COL27A1, FB_SERPINE1 and FB_COMP were involved in most interactions (Fig. 9B).

Then, we stratified these interactions for each of the molecular subtypes. Specifically, we applied CellphoneDB to 4 tumours, each representing one molecular subtype defined by bulk RNA sequencing (patient P1, P2, P5 and P6, respectively labelled by ConsensusOV as proliferative, differentiated, immunoreactive and mesenchymal types of HGSTOC) (Fig. 8A). We identified 39,979 significant reactions in the proliferative tumour (P1), 36,254 in the differentiated tumour (P2), 48,982 in the immunoreactive (P5) and 17,462 in the mesenchymal tumour (P6) (Additional file 14: Table S12). Subsequently, we assessed which major cell types were characterised by the highest number of interactions (Fig. 9C). Although fibroblasts contributed to half of the interactions found across the molecular subtypes, > 2 out of 3 interactions in the mesenchymal subtype involved one or more fibroblast subclusters. Moreover, for every cell type, the fraction of interactions involving fibroblasts was remarkably higher in the mesenchymal subtype, confirming the dominant role of fibroblasts in the mesenchymal HGSTOC.

Next, we investigated the specific interactions between cellular phenotypes across the molecular subtypes. We noticed for the proliferative subtype strong interactions between CAFs (FB_COMP, FB_COL27A1 and FB_SERPINE1) and tumour subcluster Tum_KRT17 (Fig. 9D), characterised by canonical Wnt (*Wnt5A-FZD2/FZD3/FZD5*), fibroblast growth factor (*FGF7-FGFR2/FGFR3*, *FGFR1-FGF9/NCAM1*) and transforming growth factor β (*TGFB3-TGFBR2/TGFBR3*) signalling, all promoting tumour growth. Beside the high prevalence of interactions involving fibroblasts and endothelial cells, the immunoreactive tumour was, in contrast to the other 3 molecular subtypes, characterised by a high number of interactions involving CD8+ effector memory cells (TC_GZMK), early M1 macrophages (M_CCR2) and tumour-associated macrophages (M_CCL18). For instance, *CXCL13*, a chemokine C-X-C motif ligand, known to orchestrate cell-cell interactions that regulate lymphocyte infiltration within the tumour microenvironment, was exclusively expressed by all the abovementioned immune cells in the immunoreactive patient and interacted with *ACKR4* expressed by CAFs (FB_COMP and FB_COL27A1) and the tumour-associated macrophages (M_MMP9). In the mesenchymal subtype, interactions were dominated by fibroblasts and endothelial cells. Especially pericytes (FB_RGS5), tip ECs (EC_ESM1) and capillary endothelial cells (EC_CA4) interacted more frequently compared with other molecular subtypes, demonstrating the dynamic relationship between these two cell types in promoting vessel sprouting via NOTCH signalling (*NOTCH1-DLL4*, *NOTCH4-DDL4*). Moreover, the capillary endothelial cells showed reciprocal interactions involving the vascular endothelial growth factor pathway (*VEGFA-KDR/FLT1*, *NRP1/NRP2-VEGFA/VEGFB*). Finally, in the differentiated HGSTOC patient, adipogenic fibroblasts (FB_CFD) exhibited the most interactions with ECs, especially the tip cells (EC_ESM1) and the high endothelial venules (EC_ACKR1). These cells showed a lot of interactions involving members of the tumour necrosis factor receptor superfamily (*LTBR-LTB*, *LTA-TNFRSF1A/ TNFRSF1B*, *TNFRSF1A-GRN*) regulating lymphoid organogenesis and supporting an efficient immune response and apoptosis through activation of the TNFα-κβ pathway [94] (Fig. 9D). Full list of interactions can be found in Additional file 14: Table S12.

Finally, since each molecular subtype was enriched for a specific tumour microenvironment consisting of specific cellular phenotypes, we explored to what extent the molecular subtypes represented an independent prognostic factor after correction for these 6 stromal phenotypes. To this extent, we correlated the molecular subtypes (ConsensusOV) with OS and repeated the cox regression model using FIGO stage, debulking status (residual disease), age and the 6 cellular phenotypes (by adding their respective SSZ scores to the model) as covariates. None of the molecular subtypes remained prognostic (all *p* values > 0.05, Additional file 15: Table S13), confirming that our stromal phenotypes explain, at least, part of the association of the molecular subtypes with outcome. Interestingly, when repeating the cox regression model, investigating the effect of the 6 prognostic phenotypes while adding the ConsensusOV molecular subtype labels as a covariate, only the mesothelial cells (FB_CALB2) remained significant (*p* value 0.04), albeit not surviving correction for multiple testing (BH-adjusted *p* value 0.26, Additional file 16: Table S14).

## Discussion

Our scRNA-seq study showed the phenotypic heterogeneity of HGSTOC by identifying 11 cancer cell and 32 stromal cell subtypes originating from both the primary ovarian tumour and its metastatic peritoneal or omental lesions. The cancer cell subtypes showed a large degree of patient specificity which is in line with recent work published by Izar et al. [29], who investigated the expression of 6 types of EPCAM+ tumour cells from ascites of HGSTOC patients, as well as with multiple single-cell analyses on other epithelial cancer cells [95, 96]. This patient-based clustering of tumour cells can be attributed to the unique genetic aberrations that accumulate in each individual tumour. Nevertheless, several of our cancer cell subclusters also exhibited similarity to the tumour cell subclusters identified by Izar et al. [29], suggesting that at least some common oncogenic pathways responsible for development and maintenance of HGSTOC cancer cells exist. For example, by Izar et al. identified subclusters with high expression of either *KRT17*, *KRT6A* and *MMP7* which is comparable with our subclusters TUM_KRT17, TUM_KRT6A and Tum_GJA5 respectively. Furthermore, the fallopian tube secretory epithelial cell is gaining interest, as it represents the most important cell of origin in HGSTOC. Lawrenson et al. [97] recently investigated the transcriptomic profiles of ovarian surface epithelial cells and fallopian tube secretory epithelial cells and compared them to bulk RNA profiles of HGSTOC, confirming that most of the HGSTOC derived from the latter. Furthermore, Dinh et al. [98] performed single-cell RNA sequencing on fallopian tube specimens of 8 healthy patients and found a population of early secretory cells of which transcriptomic profile was maintained in advanced HGSTOC tumours. Interestingly, a lot of the proposed genes describing this early secretory cell subcluster (e.g. *SOX17*, *PAX8*, *CRISP3*, *THY1*, *EPCAM*, *NR2F2*) were enriched in our tumour subclusters Tum_MAGEC2, Tum_KRT17, Tum_KRT6A, Tum_GJAH5, Tum_TNNT2 and Tum_CRISP3, suggesting the fallopian tube to be the cell of origin for these subclusters of tumour cells.

Remarkably, cells from Tum_KRT17 were derived from both the primary and metastatic localisation. Although intratumour heterogeneity has been extensively described in ovarian cancer [7, 8, 99], the latter findings suggest that cancer cells at different disease sites show similar transcriptomic profiles and that intratumour heterogeneity, at least partially and in a treatment-naive setting, is caused by differences in stromal cells. Indeed, several stromal components (DC_CD207, M_MMP9) showed an enrichment in a particular tissue. However, we did not analyse ovarian and omental tissue from every patient, thereby making it difficult to draw strong conclusions and underscoring the need for further analysis to confirm this hypothesis.

The current sequencing experiment from different tumour sampling sites enabled the detection of tissue-specific cells, such as Langerhans-like dendritic cells (DC_CD207) and lipid-associated macrophages (M_MMP9) enriched in the omentum. Langerin (CD207) expressing dendritic cells are indeed residing in healthy omental tissue [65] and the gut [100] where they play a role in phagocytosis. Jaitin et al. [66] performed scRNA-seq on human omental adipose tissue and also identified lipid-associated macrophages as the most strongly expanded immune cell subset in adipose tissue. Here, M_MMP9 macrophages expressed several of these adipose tissue-specific genes (e.g. *CD36*, *FABP4*, *FABP5*) as well as *TREM2*, which is involved in phagocytosis, lipid catabolism and secretion of pro-inflammatory mediators [66]. Interestingly, Olalekan et al. [30] recently characterised 9885 omental cells from 6 ovarian cancer patients and correlated the abundance of several myeloid populations (monocytes and macrophages) to high omental disease burden.

Although we only sequenced cells from 7 HGSTOC patients, we are confident that our study provides a comprehensive overview of the HGSTOC microenvironment, identifying most of the common stromal phenotypes underlying HGSTOC. Indeed, we observed a large overlap with the stromal phenotypes described by the pan-cancer blueprint of Qian et al. [64]. Only for T cells and myeloid cells, our study lacked power to identify all the subclusters. Nevertheless, this detection ratio was in line with Izar et al. [29] and Olalekan et al. [30], who also analysed ~ 15,000 cells, identifying respectively 1 and 4 T cell subclusters and both 4 macrophage subclusters. This further stresses the need to perform more in-depth analyses on a larger number of immune cells.

Although several cell types have been suggested to influence survival in ovarian cancer [12, 101–103], we are among the first to link transcriptomic profiles of individual cell phenotypes obtained from high-resolution single-cell studies with overall survival. Based on the xCell [59] deconvolution approach and another innovative method to score presence of cell types using TM genes, we discovered 6 cellular phenotypes of prognostic relevance by retrospectively analysing bulk expression data from 1467 HGSTOC patients. Firstly, mesothelial cells FB_CALB2 were identified as a prognostic subcluster. These cells showed active EMT as well as increased expression of *IL6* and its transcriptional downstream activator *STAT3*. *IL6* has recently been identified to modulate EMT in human peritoneal mesothelial cells of patients undergoing long-term peritoneal dialysis through activation of the STAT3 signalling pathway [73] as well as in pancreatic ductal carcinoma, where it promotes tumour growth and drug resistance [104]. Interestingly, Izar et al. [29] also proposed the activation of IL6 and JAK/STAT in a subgroup of fibroblasts and HGSTOC cancer cells, suggesting it is involved in the pathogenesis of malignant ascites and drug resistance.

*MYH11*/*S100A4*-expressing myofibroblasts FB_MYH11 are expected to promote cell motility, thereby promoting metastasis and reduced survival. A correlation between *MYH11* and metastasis has indeed already been demonstrated for lung and renal cell carcinoma [105, 106]. Although the unfavourable properties of myofibroblasts have frequently been demonstrated [105–107], it is usually considered that epithelial-to-mesenchymal transition (EMT) induced by hypoxia or an active TGF-β pathway is the mechanism underlying worse prognosis [108]. However, our data did not show elevated EMT nor hypoxia in the myofibroblasts, involving other mechanisms to promote disease progression. Transforming growth factor β-driven cancer-associated fibroblasts FB_COMP also correlated with reduced survival. TGF-β pathway is involved in EMT [70, 109], tumour growth and metastasis [80] and resistance to platin-based chemotherapy [110] in ovarian cancer. The TGF-β pathway has also been shown to induce expression of *CCL21* in lymphatic endothelial cells EC_PROX1 promoting the migration of tumour cells towards the lymphatic endothelial cells in breast cancer [89]. Also in ovarian cancer, EC_PROX1 has been linked to lymphatic metastasis and tumour cell invasion by inducing EMT [111]. Interestingly, the last cell type reducing survival represented a *BAMBI*-expressing tumour cluster with a clear cell-like phenotype. Clear cell ovarian tumours represent a separate subtype of ovarian tumours that is known to exhibit a highly unfavourable prognosis. Overexpression of *BAMBI* in ovarian cancer cells was previously associated with enhanced cellular proliferation, migration and reduced apoptosis, but a significant effect on OS has not yet been reported in HGSTOC [110, 112]. Marchini et al. [110], however, identified overexpression of *BAMBI* in recurrent *versus* primary HGSTOC, suggesting that *BAMBI* could induce therapeutic resistance to platinum. In this respect, HGSTOC tumours overexpressing *BAMBI* could share features with clear cell ovarian tumours [86].

The only phenotype favouring prognosis was plasma cells BC_IGHG1_PRDM1. Although plasma cells have been associated with improved survival in lung [113], colorectal [114] and breast cancer [115], their role in ovarian cancer remains controversial [74, 116, 117]. However, Kroeger et al. [74] recently demonstrated that IgG producing plasma cells were strongly associated with presence of CD8^+^ tumour infiltrating T cells and that the latter only improved survival in the presence of memory B cells, CD4 helper cells and plasma cells. This suggests that these lymphocyte subtypes promote an antitumour microenvironment.

An obvious question is whether these prognostic effects are independent of the arrayed site (i.e. the anatomic localisation of the biopsy). This obviously is a relevant question as peritoneum, omentum and ovary tissue are characterised by different microenvironments. Indeed, if a certain cell phenotype is enriched in peritoneal or omental lesions compared to ovarian tissue, our SSZ score or xCell scores could preferentially screen for peritoneal and omental tissue which are in general linked to more advanced disease (i.e. higher FIGO stage) and poor outcome [118]. We can there hypothesise that sampling site was, at least partially, corrected for by adding FIGO stage as a covariate of the meta-analysis.

Lastly, we evaluated the distribution of both the tumour and stromal cell compartment across the 4 molecular subtypes in HGSTOC and explored the presence of molecular subtype-specific interactions using CellPhoneDB. Although their impact on prognosis has often been studied, Schwede et al. [25] recently demonstrated that they lose their prognostic value after correction for stromal cell proportion [25], hence illustrating the importance of the stromal cell biology in HGSTOC. As suggested by Schwede et al. [25], we demonstrated a strong dominance of genes expressed by CAFs in the mesenchymal subtype signature, while several tumour cell phenotypes were mostly attributed to the proliferative and differentiated molecular subtype signatures. Moreover, we demonstrated enrichment of cell phenotypes correlating with poor outcome in the molecular subtypes associated with poor outcome. *Inversely*, several genes from immune cell phenotypes (especially macrophages) were enriched in the immunoreactive subtype, which was linked to improved prognosis. These findings confirm previous observations that bulk RNA profiles from the microdissected stromal or cancer component of mesenchymal HGSTOC tissue corresponded better to respectively the mesenchymal or the differentiated molecular subtype of HGSTOC [25, 119] as well as to the observations in recent single-cell studies from Izar et al. [29] and Geistlinger et al. [120].

There are several implications related to this molecular subtype analysis. Firstly, given the sparsity of scRNA sequencing data (average gene detection rate around 15–25% in a single cell), not all markers routinely used to describe a specific molecular subtype can be used to assign a subtype. Instead, we estimated the enrichment of each single-cell subcluster in all 4 molecular subtypes and evaluated the relative contribution of each subcluster in a given molecular subtype. Secondly, acknowledging the fact that the assignment of a tumour to the immunoreactive and mesenchymal subtype is largely guided by the most abundant cell type, being either immune cells and fibroblasts respectively, in their biopsy, these tumours are providing less information about the actual cancer cell subclusters they contain. Indeed, our 6 cellular phenotypes were no longer prognostic when corrected for molecular subtype, indicating that prevalence of the cellular phenotypes codetermines the molecular subtypes. Additionally, although we believe that the detection of stromal cell phenotypes can be beneficial to predict response to targeted treatment, one could wonder about the contribution of the epithelial cell compartment and whether the latter investigation is not needed to more accurately classify HGSTOC based on the pathway activity in cancer cells.

In line with Geistlinger et al. [120], we could divide the tumour cell subclusters in two groups, one harbouring a “differentiated” profile with an active immune response (Tum_MAGEC2, Tum_TNNT2, Tum_GJA5, Tum_ST6GALNAC1, Tum_KRT6A and Tum_CRISP3) and a second, exhibiting a proliferative signature with increased EMT, hypoxia and hedgehog signalling, all contributing to the more aggressive behaviour of proliferative HGSTOC. Moreover, Geistlinger et al. [120] described more amplifications, higher ploidy and more subclonal copy number alternations in tumours with a proliferative signature as compared to tumours with a differentiated profile. They suggested that both groups represent a continuum starting with a more differentiated profile, while progressing to a more proliferative signature at the end of the timeline. Indeed, our proliferative subclusters also showed an increased number of CNVs in comparison to more differentiated tumour subclusters.

Finally, by applying CellPhoneDB, we could unravel significant interactions between cell types within a tumour biopsy and observe that the majority of the interactions involved fibroblasts and endothelial cells. Acknowledging the fact that data from 4 patients with different molecular subtypes are too scarce to stipulate strong conclusions, distinct interactions were found across the 4 molecular subtypes. This could potentially be interesting to develop novel therapeutic agents to specifically target a particular molecular subtype.

Although this study already profiled a considerably large number of single cells, future studies profiling a larger number of cells from additional patients, as well as more samples from different anatomic localisations, are needed to confirm our findings. Particularly, such studies could not only strengthen our correlations with survival, but they could address whether the site of the biopsy influences the observed prognostic effects or whether other new stromal cell or cancer cell phenotypes are also contributing to overall survival. Furthermore, besides refining the cellular phenotypes and their prognostic value, additional studies should reveal whether presence of these cellular phenotypes could also have predictive value during HGSTOC treatment. Indeed, recently targeted agents, such as antiangiogenesis, immune checkpoint inhibitors and PARP inhibitors have been added to first-line treatment with carboplatin and paclitaxel [121–123]. We anticipate that scRNA-seq can assist in identifying specific cellular phenotypes that contribute to predicting and monitoring response to these treatments.

## Conclusions

In conclusion, our single-cell analysis provides a high-resolution overview of the tumour microenvironment of HGSTOC, providing 43 new potential targets for therapy and identifying 6 cellular phenotypes of prognostic relevance. Furthermore, evaluation of molecular subtype signatures in scRNA-seq data provides insights in the stromal admixture of the established molecular subtypes in ovarian cancer. We hypothesise that similar strategies will enable discovery of predictive biomarkers, facilitating a more personalised and effective treatment of HGSTOC.

## Supplementary Information

**Additional file 1: Table S1.** Patient characteristics and sample information.

**Additional file 2: Table S2.** Single-cell RNA sequencing metrics and quality control indicators for each sample, major cell type and subcluster.

**Additional file 3: Figures S1 – S7.** Supplementary Figures.

**Additional file 4: Table S3.** List of marker genes used for major cell type classification with Entrez Gene and PMID.

**Additional file 5: Table S4.** Normalised mutual information for clustering of major cell types and cell subclusters.

**Additional file 6: Supplementary File 1.** Comprehensive biological validation of identified stromal subclusters guided by the pan-cancer blueprint (Qian et al.).

**Additional file 7: Table S5.** Functional annotation of the identified subclusters based on transcriptomic profiles provided by the pan-cancer blueprint.

**Additional file 8: Table S6.** Differential gene expression analysis and functional annotation of the 11 cancer and 32 stromal subclusters.

**Additional file 9: Table S7.** Comparative analysis of subtype annotation on mutual patients in this study and Qian et al.

**Additional file 10: Table S8.** List and distribution of the 809 transcriptomic markers.

**Additional file 11: Table S9.** Results of the meta-analysis of Cox proportional hazards regression model based on Subcluster-Specific Z-scores.

**Additional file 12: Table S10.** Results of the meta-analysis of Cox proportional hazards regression model based on the xCell enrichment scores.

**Additional file 13: Table S11.** CellPhoneDB output: list of interactions between cell phenotypes from 7 patients.

**Additional file 14: Table S12.** CellPhoneDB output: list of interactions across the 4 molecular subtypes: subgroup analysis of 4 patients.

**Additional file 15: Table S13.** Effect of molecular subtypes on survival with or without correction for 6 prognostic phenotypes: meta-analysis.

**Additional file 16: Table S14.** Effect of 6 prognostic phenotypes on survival with or without correction for molecular subtypes: meta-analysis.

## Data Availability

Raw sequencing reads of the scRNA-seq experiments have been deposited in the European Genome-phenome Archive under accession number EGAS00001004987 (EGA; https://ega-archive.org/studies/EGAS00001004987) [124]. Alternatively, a download of the read count matrix, meta data and Seurat scripts is publicly available at http://blueprint.lambrechtslab.org [125]. The publicly available data for gene expression analysis were retrieved from the R package at Zenodo.org (10.5281/zenodo.32906) [61] for the Mayo Clinic cohort and from the CuratedOvarianData Bioconductor package for the 5 other cohorts (10.18129/B9.bioc.curatedOvarianData) [60]. The count matrix of the pan-cancer blueprint data from Qian et al. [64] is available as an interactive web server at http://blueprint.lambrechtslab.org [125].

## Abbreviations

HGSTOC: High-grade serous tubo-ovarian cancer
scRNA-seq: Single-cell RNA sequencing
FIGO: International Federation of Gynaecology and Obstetrics classification 2014
UMI: Unique molecular identifiers
PCA: Principal component analysis
PC: Principal component
*t*-SNE: *t*- Distributed stochastic neighbour embedding
DGE: Differential gene expression
NMI: Normalised Mutual Information
ARI: Adjusted Rand Index
CCA: Canonical correlation analysis
CNA: Copy number aberrations
CNV: Copy number variations
GSEA: Gene Set Enrichment Analysis
MSigDB: Molecular Signatures Database
SCENIC: Single-CEll regulatory Network Inference and Clustering
AUC: Area under the curve
TM: Transcriptomic marker gene
SSZ score: Subcluster-specific z-score
HR: Hazard ratio
BH: Benjamini-Hochberg correction
DC: Dendritic cell
EC: Endothelial cell
CAF: Cancer-associated fibroblast
EMT: Epithelial-to-mesenchymal transition
TGF-β: Transforming growth factor-β
OS: Overall survival
