# Supplementary material for: High-grade serous tubo-ovarian cancer refined with single-cell RNA sequencing: specific cell subtypes influence survival and determine molecular subtype classification
Source: Genome Med. 2021 Jul 9;13:111. doi: 10.1186/s13073-021-00922-x (PMC8268616; doi:10.1186/s13073-021-00922-x)
Supplement: Supplementary file 3 — Additional file 3: Figures S1 – S7. Supplementary Figures. [file 13073_2021_922_MOESM3_ESM.pdf]

Fig. S1

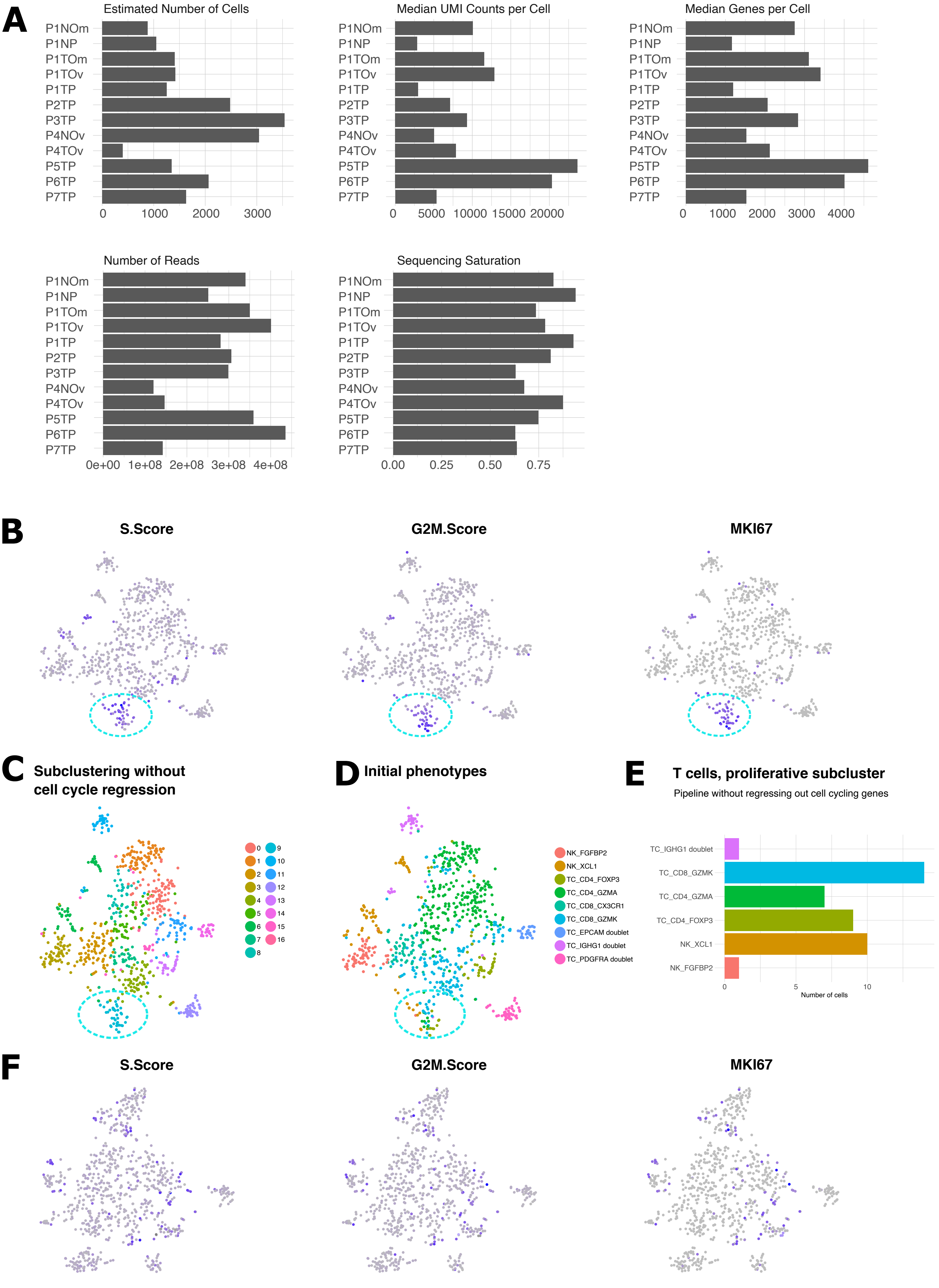
A
Estimated Number of Cells
Median UMI Counts per Cell
Median Genes per Cell
Number of Reads
Sequencing Saturation
P1NOm
P1NP
P1TOm
P1TOv
P1TP
P2TP
P3TP
P4NOv
P4TOv
P5TP
P6TP
P7TP
0 1000 2000 3000
0 5000 10000 15000 20000
0 1000 2000 3000 4000
0e+00 1e+08 2e+08 3e+08 4e+08
0.00 0.25 0.50 0.75
B
S.Score
G2M.Score
MKI67
C
Subclustering without cell cycle regression
0 1 2 3 4 5 6 7 8 9 10 11 12 13 14 15 16
D
Initial phenotypes
NK_FGFBP2
NK_XCL1
TC_CD4_FOXP3
TC_CD4_GZMA
TC_CD8_CX3CR1
TC_CD8_GZMK
TC_EPCAM doublet
TC_IGHG1 doublet
TC_PDGFRA doublet
E
T cells, proliferative subcluster
Pipeline without regressing out cell cycling genes
TC_IGHG1 doublet
TC_CD8_GZMK
TC_CD4_GZMA
TC_CD4_FOXP3
NK_XCL1
NK_FGFBP2
0 5 10
Number of cells
F
S.Score
G2M.Score
MKI67

Fig. S1 | Quality control and regression for cycling genes. A, Overview of the single-cell sequencing metrics across the different samples including the number of reads, the number of cells, average detected genes per cell, average UMIs per cell, sequencing saturation. **B,** Clustering of T cells and Natural Killer (NK) cells without regressing for cell cycle scores. t-SNE showing S phase score, G2M phase score and MKI67 expression in a proliferative cluster as highlighted by a blue circle. **C,** t-SNE visualising the new subclusters obtained without regressing for cell cycle scores. **D,** t-SNE visualising the new subclusters obtained without regressing for cell cycle scores but colour-coded for the subclusters obtained when regressing out cell cycle. **E,** Histogram showing the different T cell and NK cell subtypes in the proliferative subcluster. **F,** t-SNE of T cell subclusters after regressing out cell cycle scores, showing S phase score, G2M phase score and MKI67 expression. No distinct proliferative cluster was found.

Fig. S2

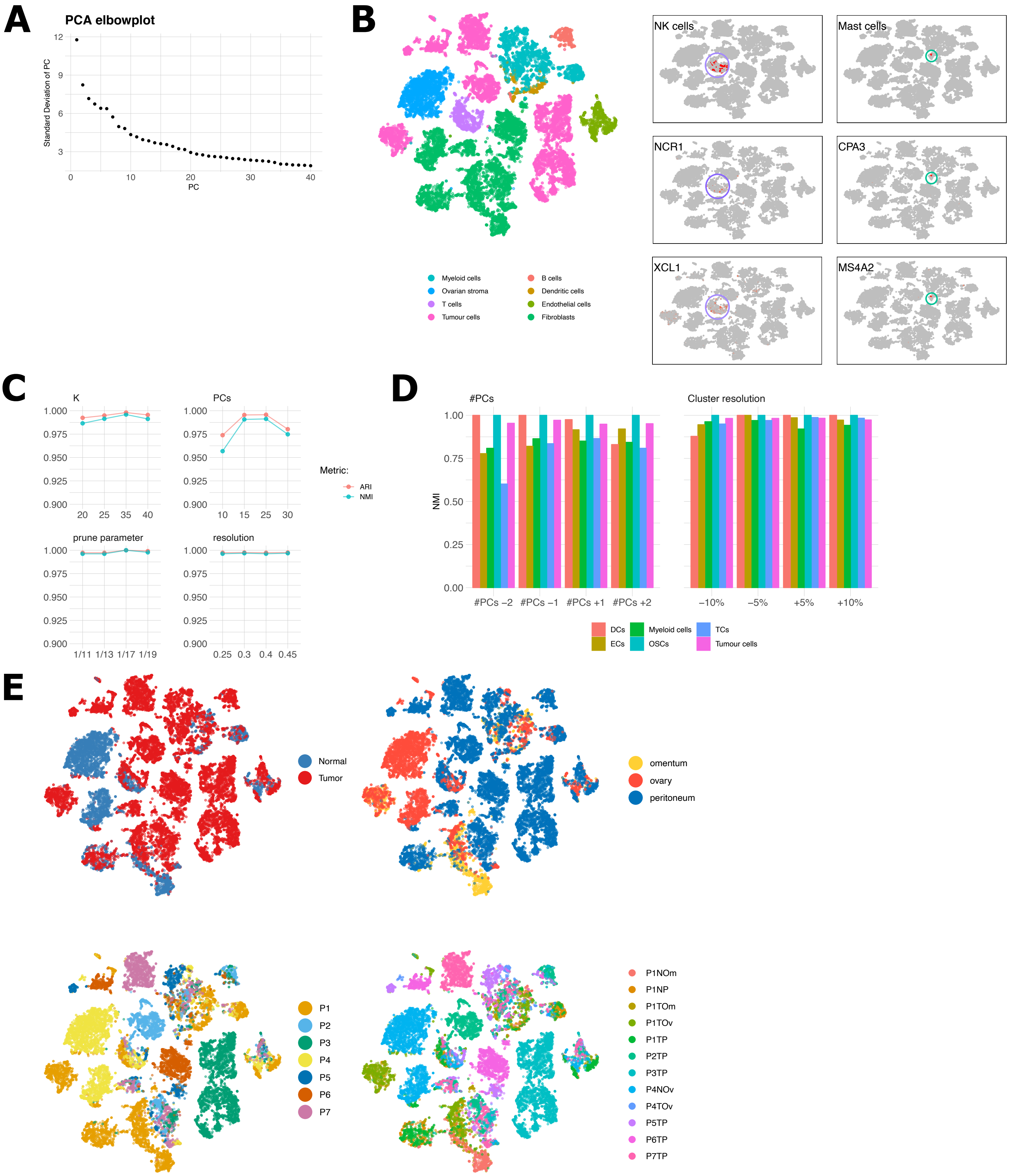
A
PCA elbowplot
Standard Deviation of PC
PC
B
NK cells
Mast cells
NCR1
CPA3
XCL1
MS4A2
Myeloid cells
Ovarian stroma
T cells
Tumour cells
B cells
Dendritic cells
Endothelial cells
Fibroblasts
C
K
PCs
prune parameter
resolution
Metric:
ARI
NMI
D
#PCs
Cluster resolution
NMI
#PCs −2
#PCs −1
#PCs +1
#PCs +2
−10%
−5%
+5%
+10%
DCs
ECs
Myeloid cells
OSCs
TCs
Tumour cells
E
Normal
Tumor
omentum
ovary
peritoneum
P1
P2
P3
P4
P5
P6
P7
P1NOm
P1NP
P1TOm
P1TOv
P1TP
P2TP
P3TP
P4NOv
P4TOv
P5TP
P6TP
P7TP

Fig. S2 | Major cell type clustering. **A,** Elbow plot analysis of principle components (PC) variance for subclustering in major cell types based on 2,766 variable genes. The curve starts to flatten around 20 PC, which was chosen as cut-off. **B**, t-SNE visualisation of the major cell types indicating the co-clustering of Natural Killers cells (*NCR1, XCL1*) and MAST cells (*CPA3, MS4A2*) with T cells and myeloid cells respectively. **C,** Robustness of cell type clustering using either lower or higher resolution, K, prune value and number of PCs. Shown is the Normalised Mutual Information (NMI)[45] and the Adjusted Rand Index (ARI)[46], between clusters of the major cell types that were generated using K of 30, the calculated number of informative PCs being 20, a resolution of 0.35 as well as a prune value of 1/15 and variations thereof. **D,** Robustness of cell subtype clustering using either lower or higher resolution or fewer or more PCs. Shown is the NMI between subclusters for each of the major cell types that were generated using the resolution and number of PCs as indicated in the manuscript (Additional file 8: Table S6) and subclusters of the same cells generated at 5% or 10% higher or lower resolution or at one or two PCs less or more. The results for fibroblasts are not shown as we used CCA-aligned subclusters. Likewise, no NMI was calculated for B cells as we deliberately chose to divide B cells in 2 main subgroups. **E,** t-SNE visualisation of all cells colour-coded for tissue specificity (normal, tumour), sampling site (omentum, ovary, peritoneum), patient (P1-P7) and sample (Sample_ID).

Fig. S3

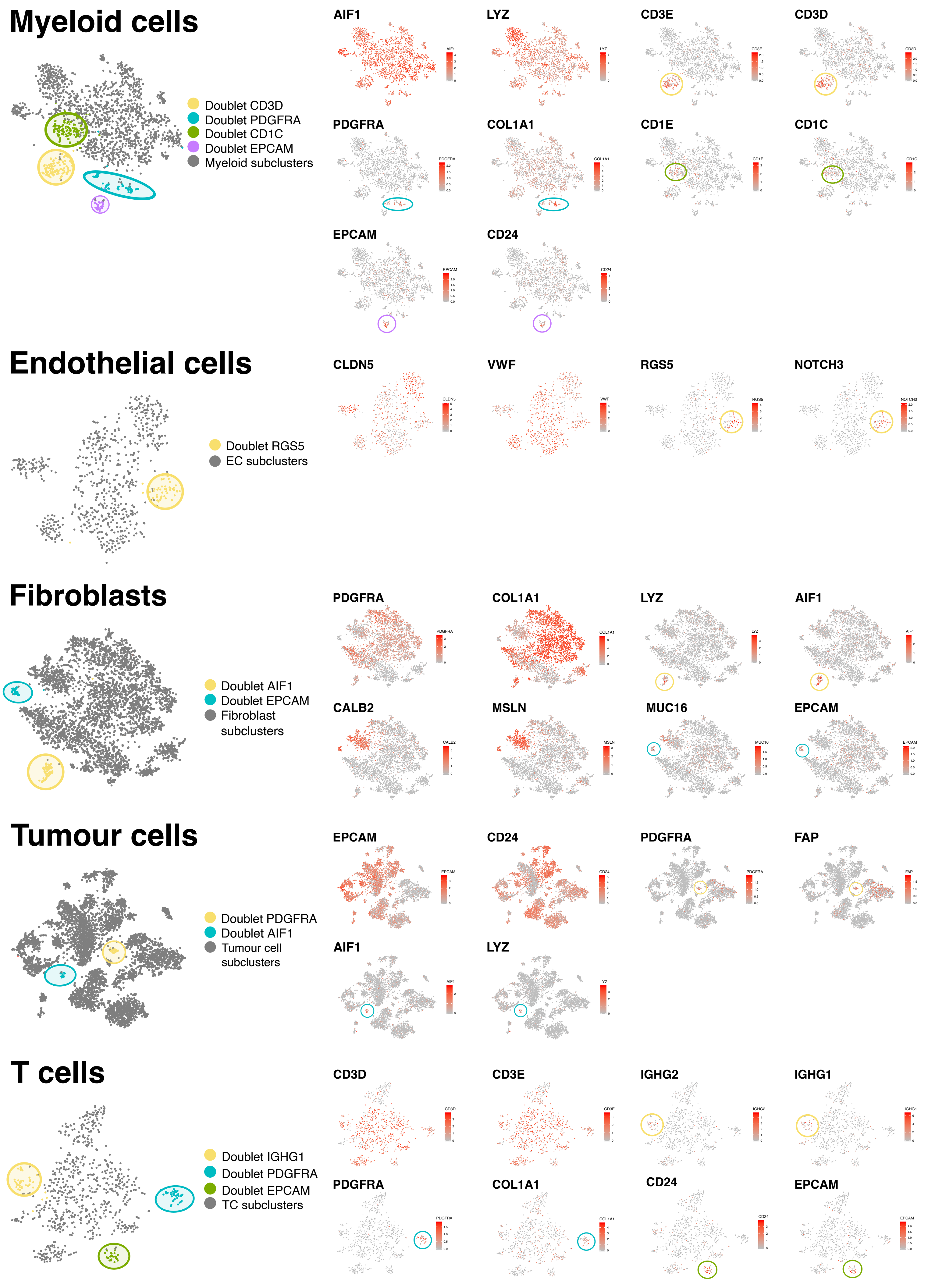

Myeloid cells
Doublet CD3D
Doublet PDGFRA
Doublet CD1C
Doublet EPCAM
Myeloid subclusters
AIF1
LYZ
CD3E
CD3D
PDGFRA
COL1A1
CD1E
CD1C
EPCAM
CD24
Endothelial cells
Doublet RGS5
EC subclusters
CLDN5
VWF
RGS5
NOTCH3
Fibroblasts
Doublet AIF1
Doublet EPCAM
Fibroblast subclusters
PDGFRA
COL1A1
LYZ
AIF1
CALB2
MSLN
MUC16
EPCAM
Tumour cells
Doublet PDGFRA
Doublet AIF1
Tumour cell subclusters
EPCAM
CD24
PDGFRA
FAP
AIF1
LYZ
T cells
Doublet IGHG1
Doublet PDGFRA
Doublet EPCAM
TC subclusters
CD3D
CD3E
IGHG2
IGHG1
PDGFRA
COL1A1
CD24
EPCAM

Fig. S3 | Detection of Doublet subclusters. t-SNE visualisation and annotation of the doublet subclusters (left) as well as the co-expression of meta genes from different major cell types (right) identified across myeloid cells, endothelial cells, fibroblasts, tumour cells and T cells. The doublet subclusters contained a combination of T cells (*CD3D, CD3E*), myeloid cells (*AIF1, LYZ*), dendritic cells (*CD1C, CD1E*), tumour cells (*EPCAM, CD24*), endothelial cells (*VWF, CLDN5*), fibroblasts (*PDGFRA, COL1A1*), pericytes (*RGS5, NOTCH3*), mesothelial cells (*CALB2, MSLN*) or B cells (*IGHG2, IGHG1*).

Fig. S4

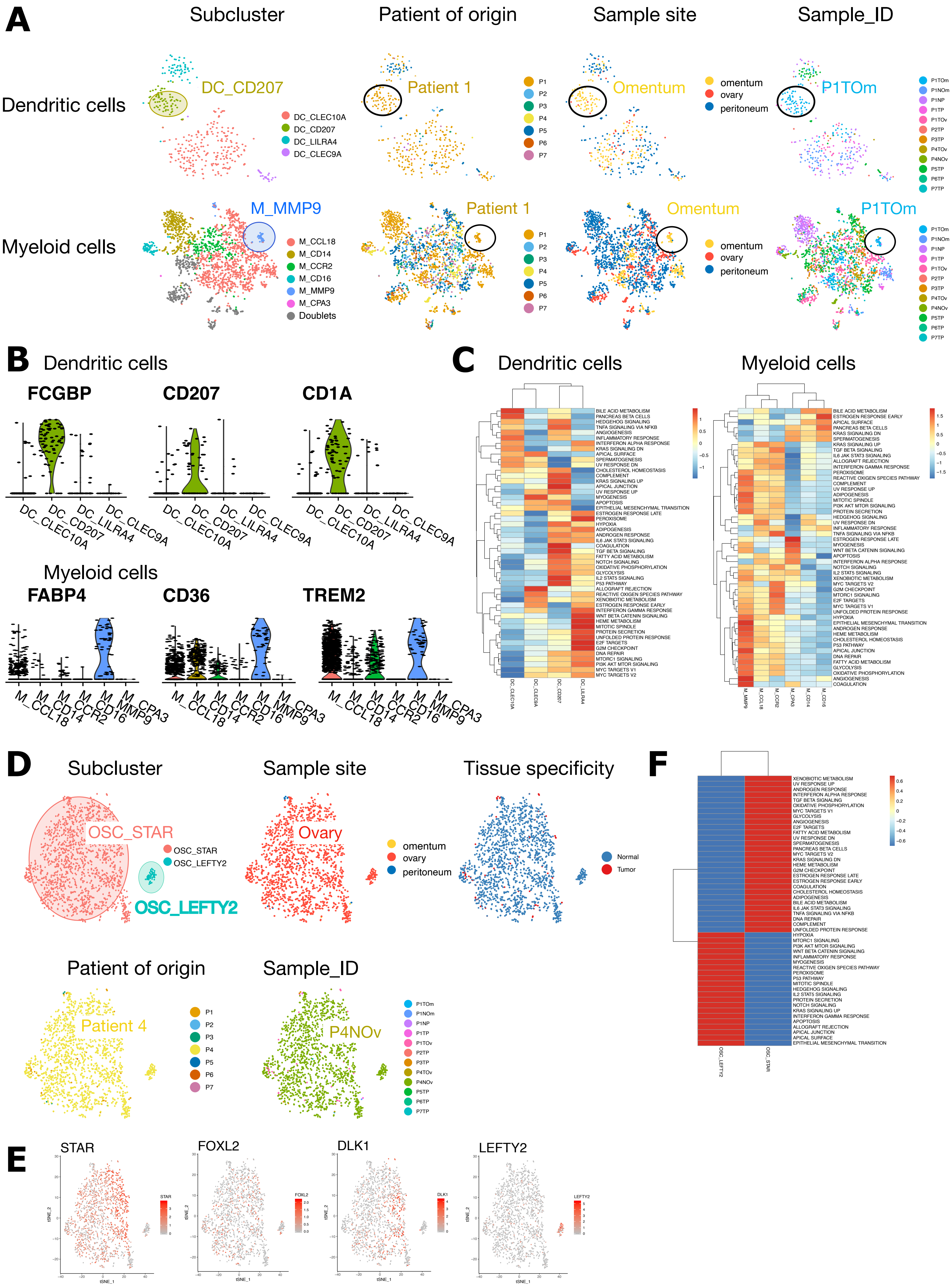
A
Subcluster
Patient of origin
Sample site
Sample_ID
Dendritic cells
DC_CD207
DC_CLEC10A
DC_CD207
DC_LILRA4
DC_CLEC9A
Patient 1
P1
P2
P3
P4
P5
P6
P7
Omentum
omentum
ovary
peritoneum
P1TOm
P1TOm
P1NOm
P1NP
P1TP
P1TOv
P2TP
P3TP
P4TOv
P4NOv
P5TP
P6TP
P7TP
Myeloid cells
M_MMP9
M_CCL18
M_CD14
M_CCR2
M_CD16
M_MMP9
M_CPA3
Doublets
Patient 1
Omentum
P1TOm
B
Dendritic cells
FCGBP
CD207
CD1A
DC_CLEC10A
DC_CD207
DC_LILRA4
DC_CLEC9A
Myeloid cells
FABP4
CD36
TREM2
M_CCL18
M_CD14
M_CCR2
M_CD16
M_MMP9
M_CPA3
C
Dendritic cells
Myeloid cells
DC_CLEC10A
DC_CLEC9A
DC_CD207
DC_LILRA4
M_MMP9
M_CCL18
M_CCR2
M_CPA3
M_CD14
M_CD16
D
Subcluster
OSC_STAR
OSC_STAR
OSC_LEFTY2
OSC_LEFTY2
Sample site
Ovary
omentum
ovary
peritoneum
Tissue specificity
Normal
Tumor
Patient of origin
Patient 4
Sample_ID
P4NOv
E
STAR
FOXL2
DLK1
LEFTY2
tSNE_1
tSNE_2
F
OSC_LEFTY2
OSC_STAR

Fig. S4 | Tissue-specific subclusters. A, t-SNE visualisation of dendritic cells (up) and myeloid cells (down) colour-coded for subclusters, patient, sample and sampling site (ovary, omentum, peritoneum). Langerhans-like dendritic cells (DC_CD207) and lipid-associated macrophages (M_MMP9) were enriched in the omentum as highlighted by a blue circle. **B,** Violin plots of marker gene expression for Langerhans-like dendritic cells (*CD1A, CD207, FCGPB*) and lipid-associated macrophages (*TREM2, CD36, FABP4*). **C,** Heatmap showing metabolic activity for dendritic (left) and myeloid cells (right). **D,** t-SNE visualisation of ovarian stromal cells colour-coded for subclusters, sampling site (ovary, omentum, peritoneum), tissue specificity (normal, tumour), patient of origin and sample. Granulosa cells (OSC_STAR) and endometrial cells (OSC_LEFTY2) were only found in non-affected ovarian tissue of patient 4. **E,** Heatmap showing metabolic activity for ovarian stroma cells. **F,** t-SNE visualisation of marker gene expression in ovarian stromal cells.

Fig. S5

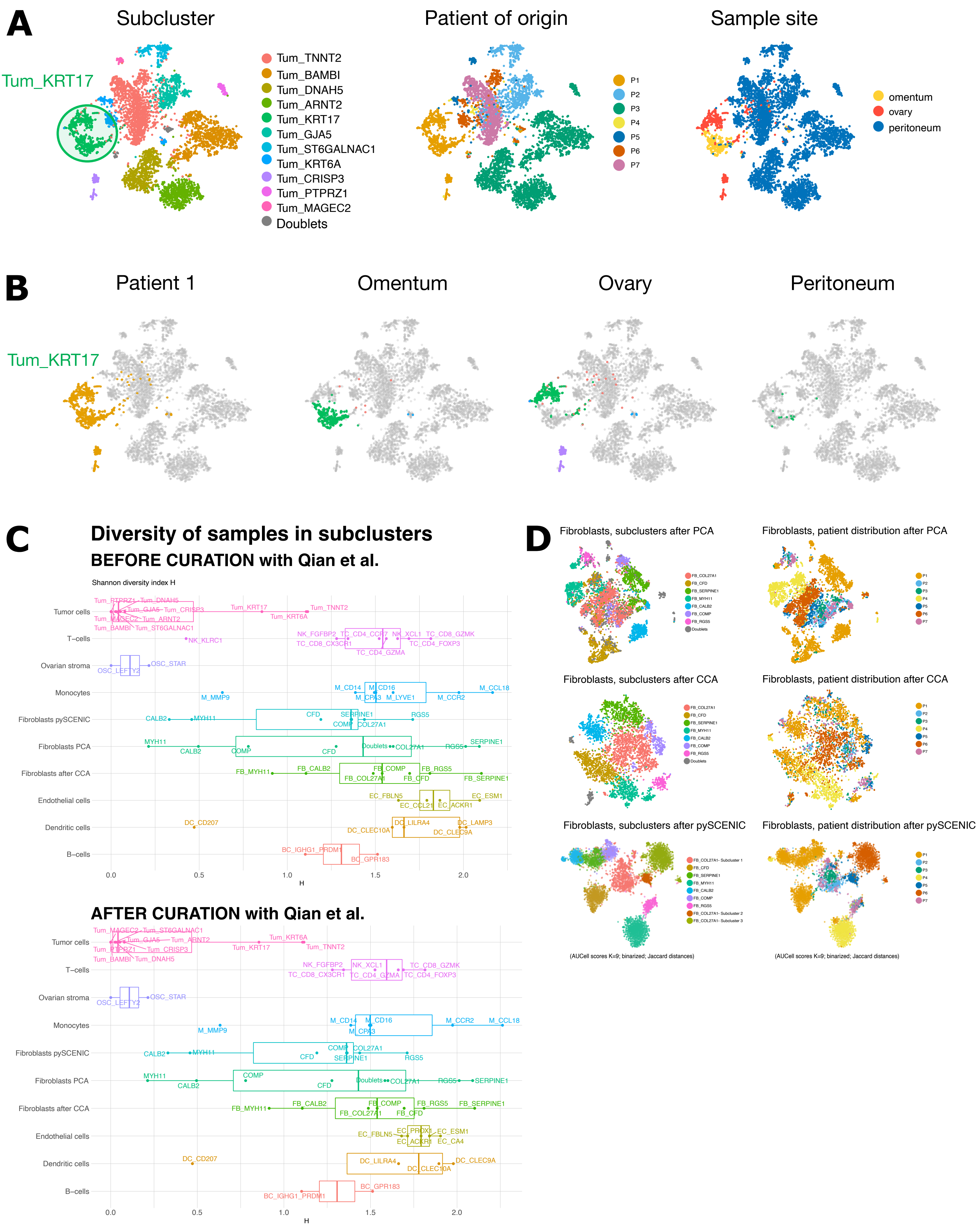
A
Subcluster
Patient of origin
Sample site
Tum_KRT17
Tum_TNNT2
Tum_BAMBI
Tum_DNAH5
Tum_ARNT2
Tum_KRT17
Tum_GJA5
Tum_ST6GALNAC1
Tum_KRT6A
Tum_CRISP3
Tum_PTPRZ1
Tum_MAGEC2
Doublets
P1
P2
P3
P4
P5
P6
P7
omentum
ovary
peritoneum
B
Patient 1
Omentum
Ovary
Peritoneum
Tum_KRT17
C
Diversity of samples in subclusters
BEFORE CURATION with Qian et al.
Shannon diversity index H
Tumor cells
T−cells
Ovarian stroma
Monocytes
Fibroblasts pySCENIC
Fibroblasts PCA
Fibroblasts after CCA
Endothelial cells
Dendritic cells
B−cells
0.0
0.5
1.0
1.5
2.0
H
AFTER CURATION with Qian et al.
Tumor cells
T−cells
Ovarian stroma
Monocytes
Fibroblasts pySCENIC
Fibroblasts PCA
Fibroblasts after CCA
Endothelial cells
Dendritic cells
B−cells
0.0
0.5
1.0
1.5
2.0
H
D
Fibroblasts, subclusters after PCA
Fibroblasts, patient distribution after PCA
Fibroblasts, subclusters after CCA
Fibroblasts, patient distribution after CCA
Fibroblasts, subclusters after pySCENIC
Fibroblasts, patient distribution after pySCENIC
(AUCell scores K=9; binarized; Jaccard distances)
(AUCell scores K=9; binarized; Jaccard distances)

Fig. S5 | Patient-specificity across the cell subclusters. A, t-SNE visualisation of cancer cell subclusters colour-coded for patient of origin, sampling site (omentum, ovary, peritoneum) and sample_ID. **B,** Zoom-in on the cancer cell subclusters originating from patient 1 as well as their distribution across the different sampling sites. **C,** Shannon indices for cell subclusters scoring sample diversity. A low Shannon index score indicates dominance of a certain sample in the subcluster while a high score indicates a more even distribution of samples/patients in the subclusters. For fibroblasts, 3 Shannon indices were visualised based on the 3 different alignments used for clustering (PCA, CCA and pySCENIC). **D,** t-SNE plot visualising the effect of the 3 different alignment methods on fibroblasts subclustering and patient-specificity.

Fig. S6

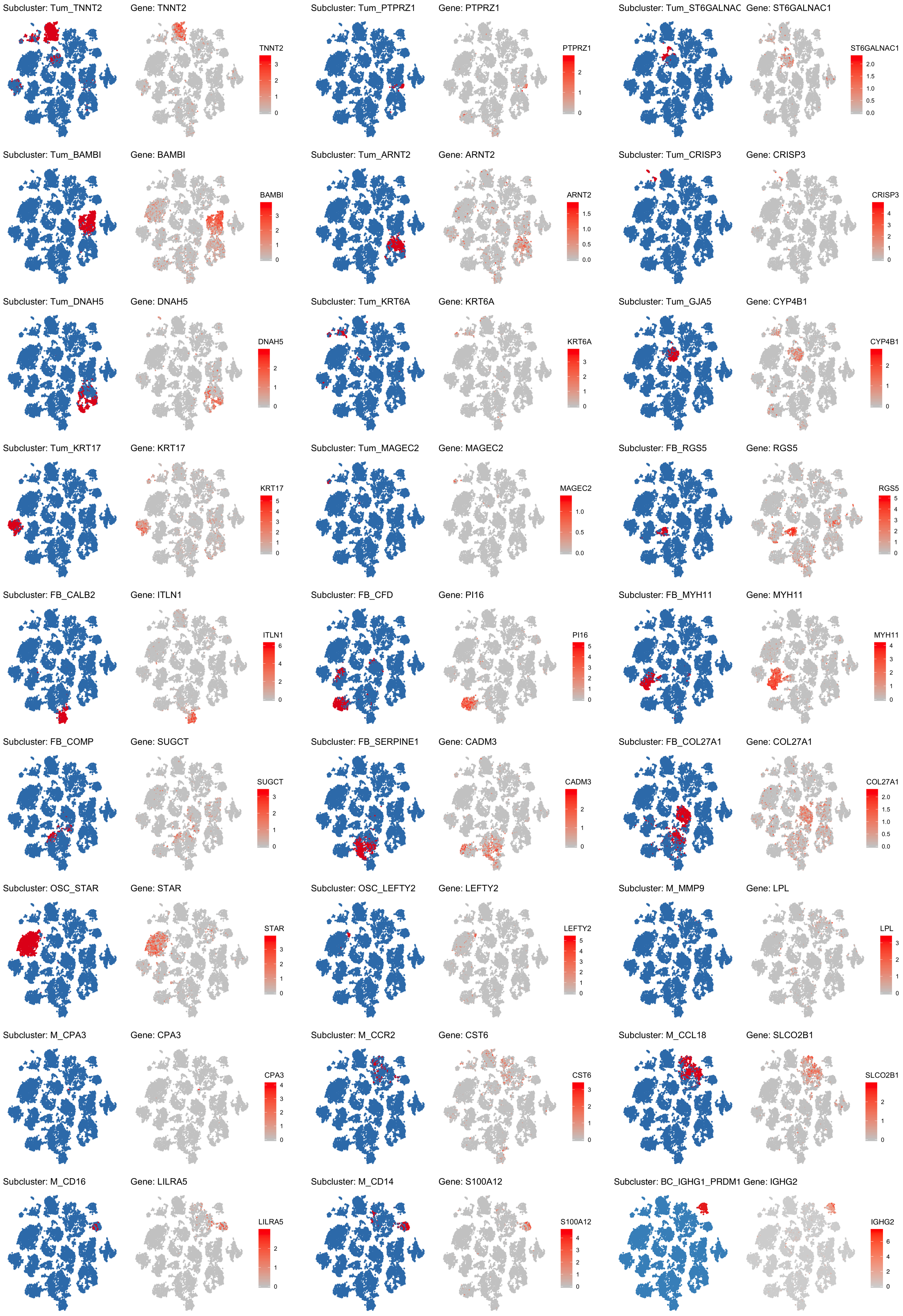

Subcluster: Tum_TNNT2
Gene: TNNT2
TNNT2
Subcluster: Tum_PTPRZ1
Gene: PTPRZ1
PTPRZ1
Subcluster: Tum_ST6GALNAC
Gene: ST6GALNAC1
ST6GALNAC1
Subcluster: Tum_BAMBI
Gene: BAMBI
BAMBI
Subcluster: Tum_ARNT2
Gene: ARNT2
ARNT2
Subcluster: Tum_CRISP3
Gene: CRISP3
CRISP3
Subcluster: Tum_DNAH5
Gene: DNAH5
DNAH5
Subcluster: Tum_KRT6A
Gene: KRT6A
KRT6A
Subcluster: Tum_GJA5
Gene: CYP4B1
CYP4B1
Subcluster: Tum_KRT17
Gene: KRT17
KRT17
Subcluster: Tum_MAGEC2
Gene: MAGEC2
MAGEC2
Subcluster: FB_RGS5
Gene: RGS5
RGS5
Subcluster: FB_CALB2
Gene: ITLN1
ITLN1
Subcluster: FB_CFD
Gene: PI16
PI16
Subcluster: FB_MYH11
Gene: MYH11
MYH11
Subcluster: FB_COMP
Gene: SUGCT
SUGCT
Subcluster: FB_SERPINE1
Gene: CADM3
CADM3
Subcluster: FB_COL27A1
Gene: COL27A1
COL27A1
Subcluster: OSC_STAR
Gene: STAR
STAR
Subcluster: OSC_LEFTY2
Gene: LEFTY2
LEFTY2
Subcluster: M_MMP9
Gene: LPL
LPL
Subcluster: M_CPA3
Gene: CPA3
CPA3
Subcluster: M_CCR2
Gene: CST6
CST6
Subcluster: M_CCL18
Gene: SLCO2B1
SLCO2B1
Subcluster: M_CD16
Gene: LILRA5
LILRA5
Subcluster: M_CD14
Gene: S100A12
S100A12
Subcluster: BC_IGHG1_PRDM1
Gene: IGHG2
IGHG2

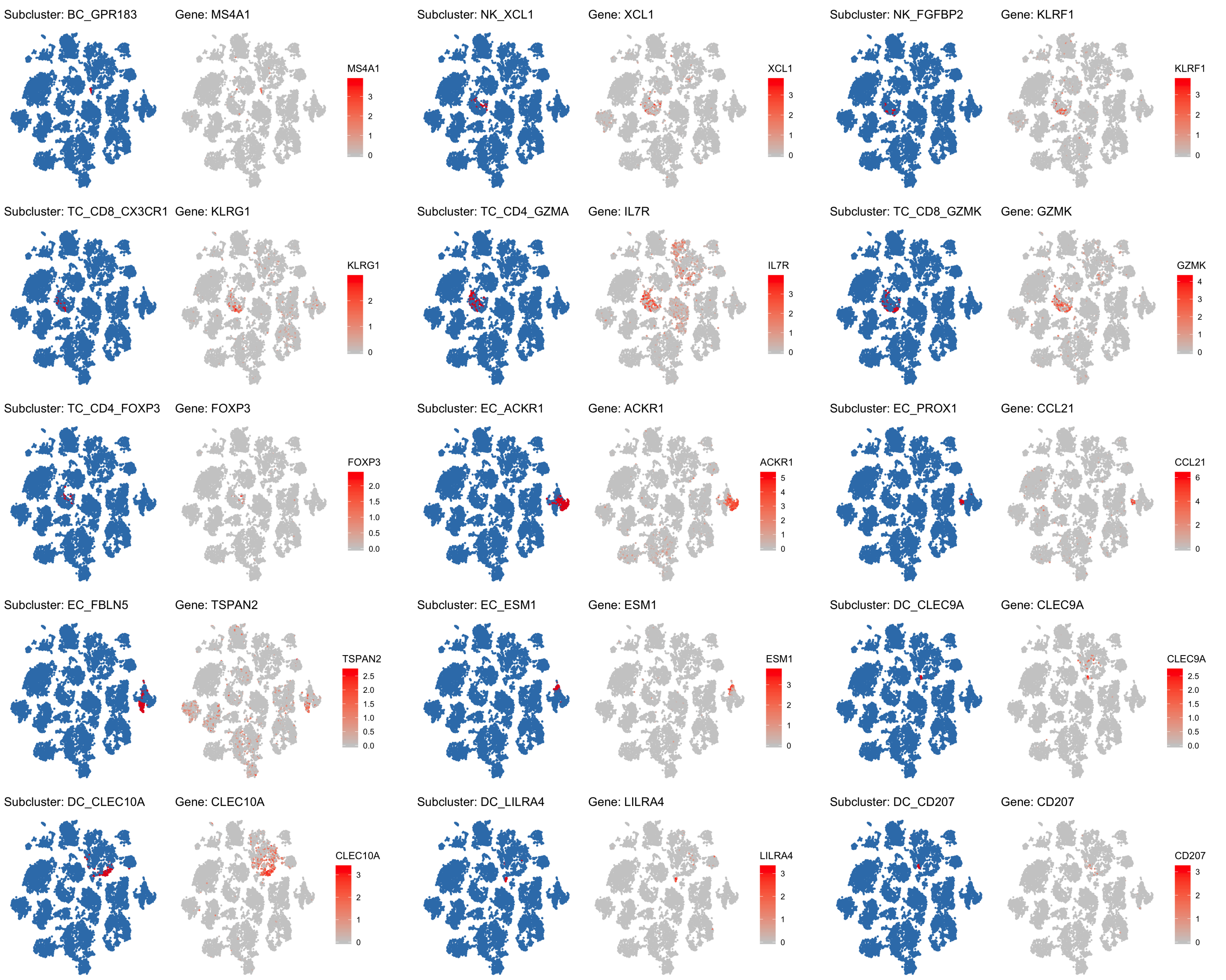

Subcluster: BC_GPR183
Gene: MS4A1
MS4A1
Subcluster: NK_XCL1
Gene: XCL1
XCL1
Subcluster: NK_FGFBP2
Gene: KLRF1
KLRF1
Subcluster: TC_CD8_CX3CR1
Gene: KLRG1
KLRG1
Subcluster: TC_CD4_GZMA
Gene: IL7R
IL7R
Subcluster: TC_CD8_GZMK
Gene: GZMK
GZMK
Subcluster: TC_CD4_FOXP3
Gene: FOXP3
FOXP3
Subcluster: EC_ACKR1
Gene: ACKR1
ACKR1
Subcluster: EC_PROX1
Gene: CCL21
CCL21
Subcluster: EC_FBLN5
Gene: TSPAN2
TSPAN2
Subcluster: EC_ESM1
Gene: ESM1
ESM1
Subcluster: DC_CLEC9A
Gene: CLEC9A
CLEC9A
Subcluster: DC_CLEC10A
Gene: CLEC10A
CLEC10A
Subcluster: DC_LILRA4
Gene: LILRA4
LILRA4
Subcluster: DC_CD207
Gene: CD207
CD207

Fig. S6 | Specificity of the 809 transcriptomic markers (TMs) defining the different subclusters (n=42/43). t-SNE visualisation of all 18,403 cells with colour-code indicating all cells belonging to one specific subcluster on the blue t-SNE and cells expressing a particular transcriptomic marker on the grey t-SNE. The subclusters, indicated on the blue t-SNE, are obtained after subclustering of each major cell type separately and then retrospectively coloured on the blue t-SNE representing all cells. On the grey t-SNE all cells, irrespective of their subcluster classification, are coloured in red according to TM expression. One subcluster of capillary endothelial cells (EC_CA4) was not shown as no specific TM was found for this subcluster.

Fig. S7

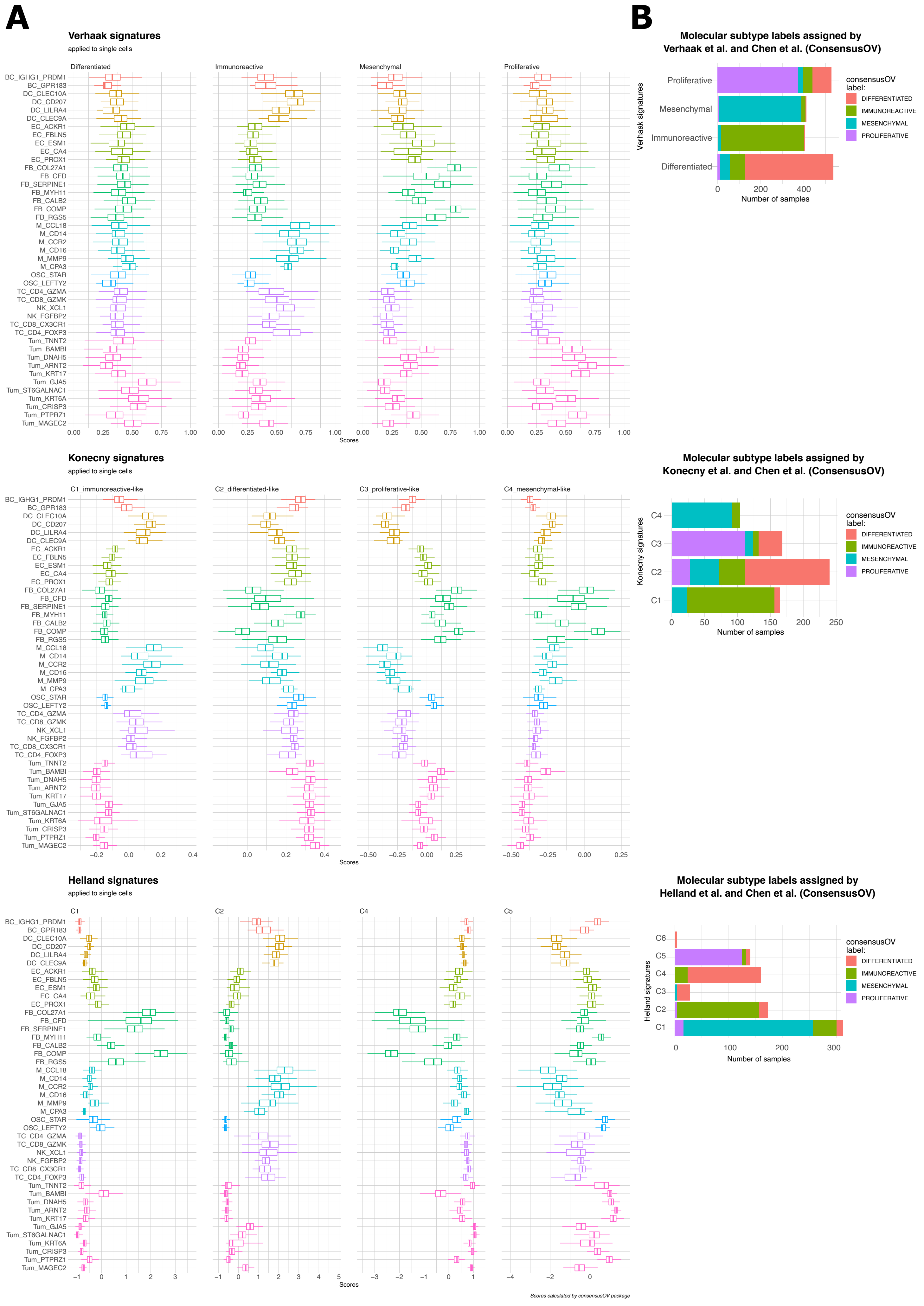

A
B
Verhaak signatures
applied to single cells
Differentiated
Immunoreactive
Mesenchymal
Proliferative
Scores
Molecular subtype labels assigned by
Verhaak et al. and Chen et al. (ConsensusOV)
Verhaak signatures
Number of samples
consensusOV label:
DIFFERENTIATED
IMMUNOREACTIVE
MESENCHYMAL
PROLIFERATIVE
Konecny signatures
applied to single cells
C1_immunoreactive-like
C2_differentiated-like
C3_proliferative-like
C4_mesenchymal-like
Molecular subtype labels assigned by
Konecny et al. and Chen et al. (ConsensusOV)
Konecny signatures
Helland signatures
applied to single cells
C1
C2
C4
C5
Molecular subtype labels assigned by
Helland et al. and Chen et al. (ConsensusOV)
Helland signatures
BC_IGHG1_PRDM1
BC_GPR183
DC_CLEC10A
DC_CD207
DC_LILRA4
DC_CLEC9A
EC_ACKR1
EC_FBLN5
EC_ESM1
EC_CA4
EC_PROX1
FB_COL27A1
FB_CFD
FB_SERPINE1
FB_MYH11
FB_CALB2
FB_COMP
FB_RGS5
M_CCL18
M_CD14
M_CCR2
M_CD16
M_MMP9
M_CPA3
OSC_STAR
OSC_LEFTY2
TC_CD4_GZMA
TC_CD8_GZMK
NK_XCL1
NK_FGFBP2
TC_CD8_CX3CR1
TC_CD4_FOXP3
Tum_TNNT2
Tum_BAMBI
Tum_DNAH5
Tum_ARNT2
Tum_KRT17
Tum_GJA5
Tum_ST6GALNAC1
Tum_KRT6A
Tum_CRISP3
Tum_PTPRZ1
Tum_MAGEC2
Scores calculated by consensusOV package

Fig. S7| Molecular subtype scores of our 18,403 single cells using other algorithms. A, All 18,403 single cells were scored individually for the presence of each molecular subtype signature using the other subtyping algorithms as described by Verhaak et al. (JCI 2013), Konecny et al. (JNCI 2014) and Helland et al. (Plos One 2011). **B,** Correlation between the molecular subtype annotation defined by the ConsensusOV signatures and Verhaak et al. (JCI 2013), Konecny et al. (JNCI 2014) and Helland et al. (Plos One 2011) respectively.
