# Supplementary material for: High-grade serous tubo-ovarian cancer refined with single-cell RNA sequencing: specific cell subtypes influence survival and determine molecular subtype classification
Source: Genome Med. 2021 Jul 9;13:111. doi: 10.1186/s13073-021-00922-x (PMC8268616; doi:10.1186/s13073-021-00922-x)
Supplement: Supplementary file 6 — Additional file 6: Supplementary File 1. Comprehensive biological validation of identified stromal subclusters guided by the pan-cancer blueprint (Qian et al.). [file 13073_2021_922_MOESM6_ESM.pdf]

Comprehensive functional annotation of identified stromal subclusters guided by the pan-cancer blueprint (Qian et al.)

AUTHORS

Siel Olbrecht[1,2,4], Pieter Busschaert[2], Junbin Qian[3,4], Adriaan Vanderstichele[1,2], Liselore Loverix[1,2,4], Toon Van Gorp[1,2], Els Van Nieuwenhuysen[1,2], Sileny Han[1,2], Annick Van den Broeck[2], An Coosemans[5], Anne-Sophie Van Rompuy[6,7], Diether Lambrechts[3,4], Ignace Vergote[1,2].

AFFILIATIONS

[1-7] Identical to main manuscript

SUMMARY

Aim To demonstrate, from a biological point of view, the robustness of the distinct stromal subclusters identified in this study we compared and curated the identified subclusters that we obtained with those identified in a recently published pan-cancer blueprint from Qian et al.[1]. Qian et al.[1] provide the biggest effort to subcluster scRNA-seq datasets across cancers thus far, including a biologically meaningful annotation for each cellular phenotype that they identified in more than 233,591 cells from 36 patients with 4 types of cancer.

Conclusion Based on the transcriptomic profiles of stromal cell types described by Qian et al.[1], 33 of our 35 subclusters showed a comparable transcriptional profile. However, a further comparison led us to merge 4 cell subclusters because a much smaller subcluster with similar expression as a much larger subcluster was found. One additional pan-cancer subcluster EC_CA4 was found by increasing the resolution of endothelial cells subclustering to 2.0. Furthermore, 2 cell subclusters did not match with a transcriptomic profile described by Qian et al., i.e. FB_COL27A1 fibroblasts and OSC_LEFTY2 granulosa cells, but were nevertheless considered as separate subclusters. Finally, 32 stromal subclusters were considered as biologically robust.

BIOLOGICAL VALIDATION IDENTIFIES 32 STROMAL CELL SUBTYPES IN HGSTOC

The recent publication of the transcriptomic profiles of 49 stromal cell types discovered in ovarian cancer by Qian et al.[1] enabled further finetuning as well as biological validation of 35 subclusters identified in our study (Additional file 7: Table S5, Sheet A). These pan-cancer blueprint profiles were yielded from 233,591 single cells from 36 patients with lung, colorectal, ovarian cancer and breast cancer, including four patients identical to this analysis (patient 1-4). Therefore, to make the phenotypic clusters of our study robust and biologically relevant, we did an exceptional effort to compare and, if necessary, curate our subclusters based on the marker gene sets from Qian et al.[1].

First, we analysed marker genes, identified in each of the 49 subclusters from Qian et al.[1], in each of the subclusters independently identified in our 7 patients (*Figure 1*). Remarkably, 33 of the 35 cell phenotypes showed a comparable transcriptional profile, despite the fact that both studies performed independent clustering with either three additional patients (patient 5-7) or >30 samples from 4 different cancer types (lung, colorectal, ovarian and breast cancer) and despite the significantly different amount of cells analysed (18,403 vs. 233,591 cells respectively). Interestingly, among these 33 commonly identified phenotypes, we detected 4 cell phenotypes representing conventional dendritic cells type 2 (DC_CLEC10A; 216 cells), tumour-associated macrophages (M_CCL18; 930 cells), CD4+ effector-memory cells (TC_CD4_GZMA; 200 cells) and chemo-attractant NK cells (NK_XCL1; 51 cells) for which each time a second, much smaller subcluster was considered with similar expression of the marker genes as identified by Qian et al.[1], namely DC_LAMP3 (56 cells), M_LYVE1 (140 cells), TC_CD4_CCR7 (69 cells) and NK_KLCR1 (18 cells) (*Figure 1*). Acknowledging that the transcriptomic profiles of the pan-cancer blueprint were based on considerably more cells — hence being more robust and less influenced by technical errors (e.g. resolution artefacts)[2], we decided to merge these cell phenotypes with their larger counterpart.

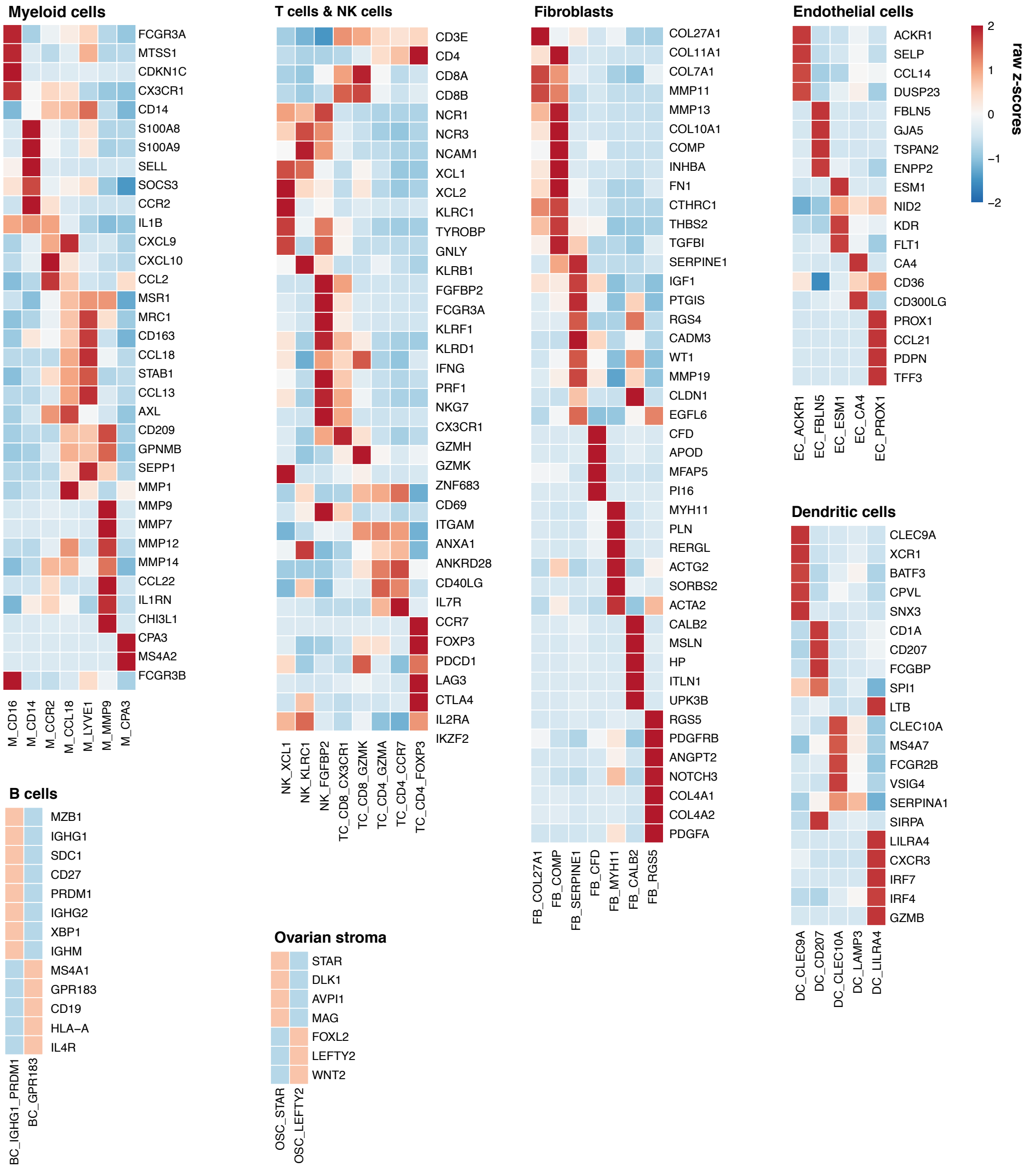

Figure 1: *Heatmap showing expression of all marker genes identified by Qian et al.[1] applied to the 35 stromal subclusters identified in this study.*

In our 35 subclusters, we did not retain an individual subcluster containing capillary endothelial cells (ECs). Based on the established marker genes (*CA4, CD300LG*) defined by Qian et al. we were also able to distinguish a small subgroup of capillary ECs (*Figure 2)* originating from the high endothelial venules (EC_ACKR1) subcluster. This is not surprising as *CA4* inhibits the capillary-like organisation of human venous ECs[3]. By augmenting the resolution from 0.5 to 2.0, we were able to separate this subcluster of capillary ECs. As the detection of this subtype

of endothelial cells is of biological importance, we considered this as a separate cellular subcluster.

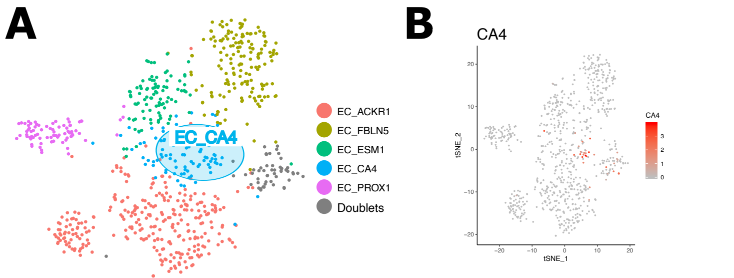

Figure 2: A) *t-SNE visualisation of the endothelial cell subclusters at resolution 2.0.* ***B)*** *t-SNE visualisation of the endothelial cells with the expression of the capillary EC marker CA4.*

Two cell phenotypes did not match with a transcriptomic profile described by Qian et al. [1], in particular, FB_COL27A1 and OSC_LEFTY2. While Qian et al.[1] identified 2 types of cancer-associated fibroblasts defined by FB_SERPINE1 and FB_COMP, we found an additional cancer-associated fibroblast subcluster FB_COL27A1 with intermediate *COL10A1* and *COL11A1* expression but low expression of *SERPINE1* and *COMP* (*Figure 1*). Instead, these cells showed an upregulation of other collagens (*COL27A1*) and metalloproteases *MMP11* and *MMP13*, suggesting a role in extra cellular matrix remodelling (*Figure 3*)[4]. Based on the large number of cells in this subcluster and the tendency of fibroblasts to be driven by cancer/tissue-specific factors, we decided to include this cluster in our downstream analysis as a separate and additional subcluster.

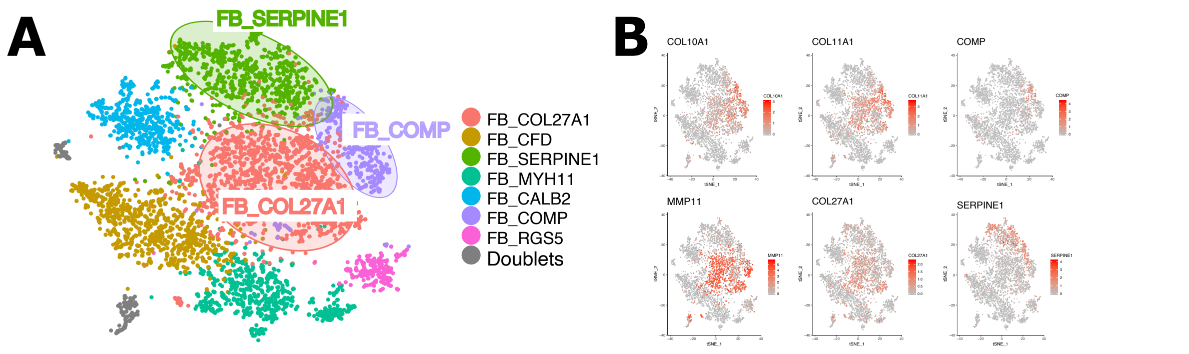

Figure 3: A) *t-SNE visualisation of the cancer-associated fibroblasts in our study colour-coded for the different subclusters and* ***B)*** *the marker gene expression for these subclusters.*

Furthermore, in contrast to the pan-cancer blueprint paper in which ovarian stromal cells were identified as a subcluster of ovarian-specific fibroblasts, we here separated these ovarian stromal cells from the other fibroblasts on a cell type level and performed independent

subclustering for both major cell types. The underlying reason to analyse stromal ovarian cells separately, was a low expression of fibroblast marker genes (*BGN, COL1A2, COL1A2*) while these cells expressed *STAR* and *FOXL2*, known as markers of granulosa cells (main manuscript Fig.1B)[5]. Subsequent separate subclustering divided these "granulosa cells" not only in a large subcluster (OSC_STAR) representing the actual granulosa cells with similar marker genes as their pan-cancer counterpart (FB_STAR_CAF and FB_STAR_NF), but also in a smaller subcluster of cells characterised by the expression of *LEFTY2,* indicative for the presence of human endometrial stromal cells as described in the main manuscript[6].

Interestingly, some cell phenotypes, described as shared across cancer types by Qian et al.[1], were, for several reasons, not identified as a separate subcluster in our analysis (Additional file 7: Table S5, Sheet A). <u>Firstly</u>, classification of B and mast cells was predominantly guided by other cancers as the abundance of the B cells and mast cells analysed by Qian et al.[1] was found in lung and colorectal cancer (Additional file 7: Table S5, Sheet B). <u>Secondly</u>, neutrophils and migratory dendritic cells are rare in HGSTOC, even in large study as the pan-cancer blueprint[1] including only 13 and 9 ovarian-cancer derived neutrophils and migratory DCs respectively (*Figure 4*) (Additional file 7: Table S5, Sheet C). Of note, neutrophils are also known to contain a low number of RNA transcripts per cell, hence rendering their detection by scRNA-seq difficult.

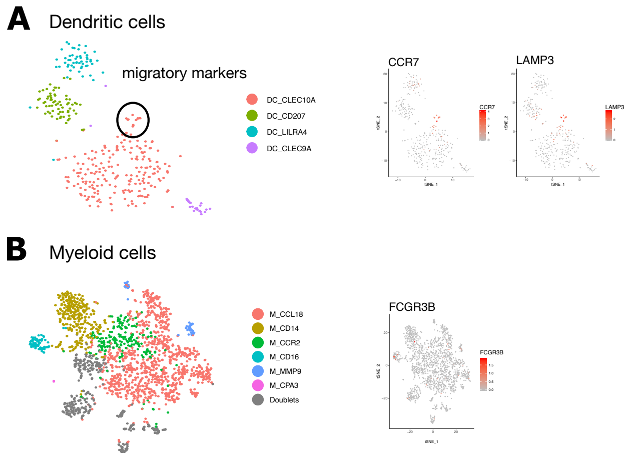

Figure 4*: A-B, t-SNE plot of all dendritic cells (A) and myeloid cells (B) analysed in this study with the expression of migratory markers (CCR7, LAMP3) and neutrophil markers (FCGR3B) as published by Qian et al.*

Thirdly, especially for highly plastic cell phenotypes like T cells and macrophages, this study did not provide the adequate number of cells to discriminate closely-related cells (Additional file 7: Table S5: Sheet D). For example, our effector memory population TC_CD8_GZMK and TC_CD4_GZMA was mixture of cells with a more naive (*CCR7, IL7R*), a more resident memory (*ZNF683, CD69*) and to a small extent even exhausted T cell (*HAVRC2, CTLA4, PDCD1, CXCL13)* profiles (*Figure 5*). However, a close link between these phenotypic states has already extensively been reported[1,7] and since they were difficult to separate even when using increased resolution, we decided not to further separate this cluster into additional small subclusters.

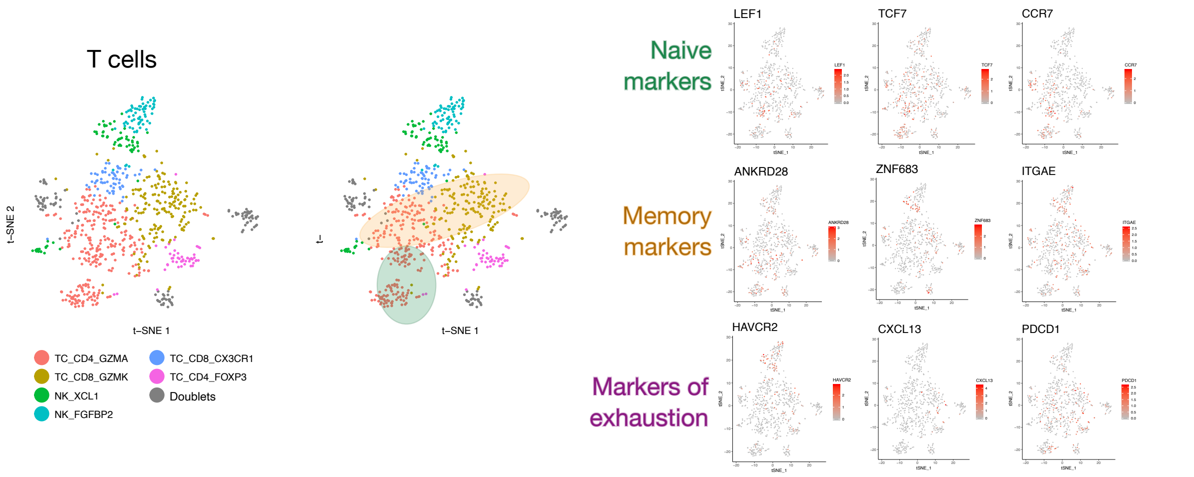

Figure 5: *t-SNEs of T cells colour-coded for each separate T cell subcluster as well as the expression of marker genes as described by Qian et al.[1] demonstrating the diffuse expression of naive markers (LEF1, TCF7, CCR7), memory markers (ZNF683, ANKRD28, ITGAE) and, albeit to a lesser extent, exhaustion markers (PDCD1, HAVCR2, CXCL13) in the effector memory CD4+ and CD8+ effector memory T cells respectively.*

Likewise, macrophages with an intermediate inflammatory profile (*CCL2*) were identified among the early macrophages (M_CCR2), although at the interface of M_CCR2 and the M2 macrophages (M_CCL18) (*Figure 6*). Based on the rather pro-inflammatory function of both cell phenotypes as well as their developmental connection, these subclusters can indeed be merged from a biological point of view. Similarly, we only identified two subclusters of

tumour-associated macrophages, M_MMP9 and M_CCL18. Based on the pan-cancer profiles, we identified a subgroup of cancer-associated macrophages (*CX3CR1, CCL3*) and a subgroup of perivascular M2 macrophages (*LYVE1, EGFL7*) in the borders of M_CCL18 (*Figure 6*). Nevertheless, as they all showed an increased expression of M2 genes, we kept all these cells in one M_CCL18 cluster, based on their functional coherence.

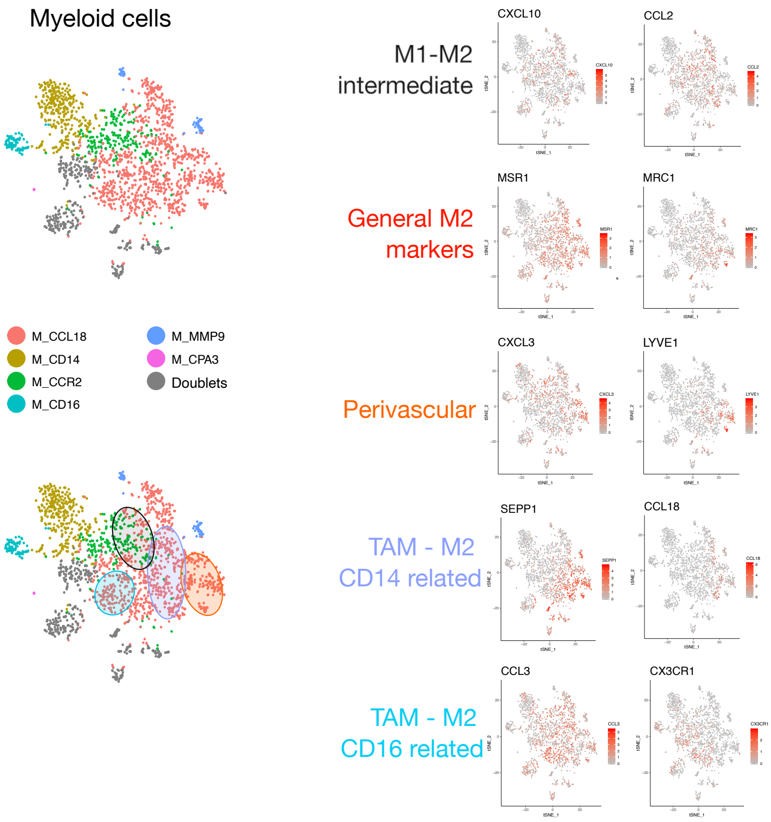

Figure 6: *t-SNE visualisation of myeloid cells colour-coded for subclusters as well as for the expression of marker genes described by Qian et al.[1] defining subgroups of intermediate macrophages (CCL2, CXCL10) at the interface of M_CCR2 and M_CCL18 as highlighted by the black circle, a subgroup of perivascular macrophages (LYVE1, CXCL3) indicated by the orange circle and two distinct categories of tumour-associated macrophages either being CD14- (CCL18, STAB1) or CD16-related (CX3CR1, CCL3), highlighted by a purple and blue circle respectively. As the latter three groups show an increased expression of M2 genes, we kept all these cells co-clustered.*

In the end, 31 of the initial 35 stromal subclusters as well as the additional subcluster of capillary endothelial cells EC_CA4 were considered to be biologically robust and were retained for further downstream analysis. Based on the similarity of the final 32 subclusters, we also standardised all subcluster labels in accordance with Qian et al.[1]. Full functional annotation of these subclusters, the parameters to obtain these subclusters (number of variable genes, PCs,

resolution) to obtain these subclusters as well as the differential gene expression analysis can be found in Additional file 8: Table S6.

Finally, we performed a subgroup analysis on the 4 patients included in both studies and counted the cells with an identical annotation in both studies (Fig. 2B–C, main manuscript). Remarkably, 98.5 % of cells were attributed to the same major cell type, being the lowest for dendritic cells (90.4%) and myeloid cells/mast cells (94.3%). This high overlap in major cell type annotation was confirmed by a normalised mutual information of 0.94. An estimation of the number of cells assigned to the same cell phenotype as in Qian et al[1] was less straightforward because not all subclusters were present in both analysis as described above. Nevertheless, after 1) merging the blueprint B cell subclusters into either follicular or plasma cells, 2) merging our two types of ovarian stroma cells and 3) acknowledging the presence of biologically related cell states in one and the same T cell and macrophage subclusters, we obtained an identical annotation for 85.6% of the cells with, as expected, the lowest identical subcluster annotation for T cells (75.2%) and myeloid cells (82.4%). As expected, the normalised mutual information showed a slightly lower value of 0.83, still showing a strong overlap in subcluster annotation. A detailed overview of shared cell distribution as well as the individual NMIs per major cell type can be found in Additional file 9: Table S7.

ADDITIONAL REFERENCES

1. Qian J, Olbrecht S, Boeckx B, Vos H, Laoui D, Etlioglu E, et al. A pan-cancer blueprint of the heterogeneous tumor microenvironment revealed by single-cell profiling. Cell Res. 2020;30(9):745-762.

2. Jindal A, Gupta P, Jayadeva, Sengupta D. Discovery of rare cells from voluminous single cell expression data. Nat Commun. 2018;9:4719.

3. Su M, Huang J, Liu S, Xiao Y, Qin X, Liu J, et al. The anti-angiogenic effect and novel mechanisms of action of Combretastatin A-4. Sci Rep. 2016;6:1–11.

4. Arolt C, Meyer M, Hoffmann F, Wagener-Ryczek S, Schwarz D, Nachtsheim L, et al. Expression profiling of extracellular matrix genes reveals global and entity-specific characteristics in adenoid cystic,

mucoepidermoid and salivary duct carcinomas. Cancers (Basel). 2020;12:1–18.
5. Georges A, Auguste A, Bessiere L, Vanet A, Todeschini A-L, Veita RA. FOXL2 : a central transcription factor of the ovary. J Mol Endocrinol. 2014;52:17–33.
6. Salker MS, Singh Y, Durairaj RRP, Yan J, Alauddin M, Zeng N, et al. LEFTY2 inhibits endometrial receptivity by downregulating Orai1 expression and store-operated Ca2+ entry. J Mol Med. Journal of Molecular Medicine; 2018;96:173–82.
7. Zhang L, Yu X, Zheng L, Zhang Y, Li Y, Fang Q, et al. Lineage tracking reveals dynamic relationships of T cells in colorectal cancer. 2018; 564(7735):268-272
